# New Contributions on Species Diversity of Genus *Hydnum* and *Lentaria* s.l. in China

**DOI:** 10.3390/jof10120824

**Published:** 2024-11-27

**Authors:** Linjie Su, Taijie Yu, Rou Xue, Wenhao Zhang, Chang Xu, Xing Xia, Jia Li, Hanchi Lei, Yang Dong, Guoli Zhang, Liping Tang

**Affiliations:** 1 School of Pharmaceutical Sciences and Yunnan Key Laboratory of Pharmacology for Natural Products, Kunming Medical University, Kunming 650500, China; suull121@163.com (L.S.); 18385037921@163.com (T.Y.); 18787089431@163.com (R.X.); zhangwenhao1@kmmu.edu.cn (W.Z.); xuchang727298@163.com (C.X.); xiaxing3790@outlook.com (X.X.); 18892800409@163.com (J.L.); 15559729789@163.com (H.L.); 2 Yunnan College of Modern Biomedical Industry, Kunming Medical University, Kunming 650500, China; 3 Forestry Bureau of Shitai County, Shitai 245100, China; 15256009347@163.com

**Keywords:** taxonomy, species delimitation, coralloid fungi, edible mushroom, spinous fungi

## Abstract

Southwest China is extremely rich in fungal resources, and a large number of new taxa have been discovered in recent years. In the present study, we examined 26 specimens of the genera *Hydnum* and *Lentaria* sensu lato, most of which were obtained in Yunnan Province. Through ITS-nrLSU-*tef1* phylogenetic analysis, combined with morphological studies and geographic analyses, five new species were described, viz. *H. cremeum* (奶油齿菌), *H. flavoquamosum* (黄鳞齿菌), *H. roseoalbum* (粉白齿菌), *H. roseotangerinum* (粉橙齿菌), and *L. subalpina* (亚高山木瑚菌). Furthermore, we also supplied new information on some known species, including host plants and new distribution records. We re-examined the holotype sequences of two known taxa, *H. flabellatum* and *H. pallidomarginatum*, treating *H. flabellatum* as a synonym of *H. pallidomarginatum*. Additionally, a key to *Lentaria* s.l. in China was provided.

## 1. Introduction

Yunnan Province, China, is located in the southwestern extension of the Himalayas. It has tropical rainforests, subtropical evergreen broad-leaved forests, and subalpine coniferous forests, which provide favorable habitats and symbiotic hosts for the growth of fungi [1,2,3]. As suggested by Hawksworth, the number of fungi (including lichens) occurring in a given area is roughly six times as high as the native plant populations, and thus, a total of approximately 104,000 fungal species would be expected in Yunnan [4]. Up to date, about 3000 species of higher fungi have been reported in Yunnan [5,6,7,8,9,10,11,12,13,14,15,16]. This means that a vast number of macrofungal resources in this region remain to be explored.

*Hydnum* L. belongs to Hydnaceae, Cantharellales, which was established by Linnaeus in 1753 and typed as *H. repandum* L. [17,18]. Species in this genus are morphologically characterized by white, ochraceous to brownish-orange basidiomata with a spinous hymenophore, often developing stipe, smooth, hyaline, globose, subglobose to ellipsoid basidiospores, and clavate to subfusiform basidia with one to eight sterigmata [17,18,19,20,21,22,23,24,25,26]. *Hydnum* is distributed worldwide and usually ectomycorrhizal fungi, which can establish symbiosis with wide ranges of host plants, such as species in Dipterocarpaceae, Fagaceae, Malvaceae, Pinaceae, and Salicaceae [18,19,21,22,23]. Previously, *Hydnum* included taxa with a spinose hymenophore, but a majority of species were later transferred to the genera *Hericium* Pers., *Phellodon* P. Karst., and *Sarcodon* Quél. Ex P. Karst. [17,18,20,22,27]. According to molecular phylogenetic results, the genus *Hydnum* was divided into five subgenera, viz. the subgen. *Alba* Niskanen & Liimat., subgen. *Brevispina* T. Cao & H. S. Yuan, subgen. *Hydnum* L., subgen. *Pallida* Niskanen & Liimat., and subgen. *Rufescentia* Niskanen & Liimat. [18,20]. The species of *Hydnum* were mainly found in temperate regions, only a few taxa were reported from subtropical to tropical forests [18,19,20,22,23,24,25,28]. Recently, new species from the genus *Hydnum* were continuously reported in China. Until 2024, 22 species have been reported in China, and seven of them were distributed in Yunnan Province [18,19,21,26,29]. However, the diversity of *Hydnum* in China is still seriously underestimated [4].

The genus *Lentaria* Corner, belonging to Lentariaceae, Gomphales, was proposed in 1950, and it was widespread from temperate to tropical zones in the world [30,31,32,33,34]. Species in the genus *Lentaria* s.l. are characterized by coralloid basidiomata emerging from a subiculum, are superficial or immersed on the substrate, often branched, with high length/width ratio basidiospores, a monomitic hyphal system, and being saprotrophic on the dead wood of various plants. This genus plays important roles in forest ecosystems [31,33,34]. However, there is little information available on it [30,31,34]. So far, only 23 records can be found in the Index Fungorum (https://www.indexfungorum.org/, accessed on 18 November 2024). Among them, five species have been reported in China, viz. *L. bambusina* P. Zhang & Zuo H. Chen, *L. byssiseda* Corner, *L. patouillardii* (Bres.) Corner, *L. surculus* (Berk.) Corner, and *L. uncispora* P. Zhang & Zuo H. Chen, with only *L. surculus* recorded in Yunnan [31]. Additionally, according to previous studies, this polyphyletic genus needs further study [31,33].

In the past years, some specimens from two genera, *Hydnum* and *Lentaria* s.l., have been collected, mainly in southwestern China. Based on both morphological and molecular data, this study aims to (i) identify and describe new taxa; (ii) supply new information for five known species from the genera *Hydnum* and *Lentaria* s.l.; and (iii) discuss one doubtful species and clarify the species diversity of *Hydnum* and *Lentaria* s.l. in China.

## 2. Materials and Methods

### 2.1. Specimen Collecting

**Figure 1:**
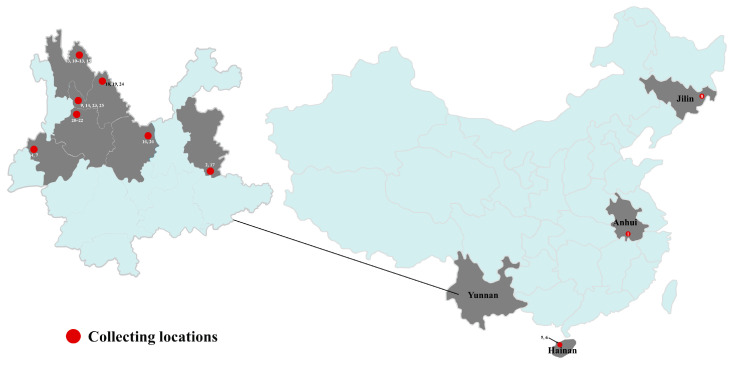
Collecting locations of specimens in China (Numbers correspond to the specimen voucher available in Table 1).

Twenty-six specimens were collected during the rainy season from Anhui, Hainan, Jilin, and Yunnan Provinces in China from 2015 to 2024. Fresh fruiting bodies were dried with a dryer at 50–60 °C and silica gel after detailed field records were made on the same day. Those collections were deposited in the Mycological Herbarium of Kunming Medical University (MHKMU). Detailed information is shown in Table 1 and Figure 1.

### 2.2. Morphological Studies

The macroscopic morphology was described based on field records and photographs of basidiomata and color codes referred to by Kornerup and Wanscher [35]. The size of basidiomata is referred to by Bas C [36]. Micro-morphological structures were observed under a Leica DM2500 light microscope (Leica Microsystems, Wetzlar, Germany) from dried materials, which were mounted in 5% potassium hydroxide (KOH) and stained with 1% Congo Red solution (*w*/*v*) when necessary. Melzer’s reagent was used to examine the amyloidity of basidiospores. In this paper, [n/m/p] refers to ‘n’ basidiospores measured from ‘m’ basidiomata of ‘p’ collections; ‘(a) b–c (d)’ refers to the length and width of basidiospores, ‘a’ and ‘d’ refer to the minimum and maximum values of measured values, and ‘b–c’ refers to the distribution interval of 90% of measured values; ‘Q’ means the length/width ratio of a basidiospore; and ‘Qm’ refers to mean ± standard deviations of Q values of all basidiospores.

### 2.3. DNA Extraction, PCR Amplification and Sequencing

Total genomic DNA was extracted from ca. 20 mg of dried basidiomata tissue using the modified CTAB method [37]. Three DNA loci, nuclear ribosomal DNA internal transcribed space (ITS) regions, large subunit nuclear ribosomal RNA (nrLSU), and translation elongation factor 1 (*tef1*) were amplified by polymerase chain reaction (PCR) using primer pairs ITS5/ITS4, LR0R/LR5, 983F/1567R, and HEF1F/HEF1R, respectively [19,38,39,40].

The PCR amplification reaction system consisted of 12.5 μL 2 × Taq PCR Master mix (Biomed, Beijing, China), 1 μL of each primer (5 μM), 1 μL template DNA, and then was refilled to 25 μL with sterilized, double-steamed water. The PCR conditions followed the description of Tang et al. [41], which were as follows: pre-denaturation at 94 °C for 5 min; 35 cycles of denaturation at 94 °C for 40 s, annealing at 56 °C for 40 s, and extension at 72 °C for 1 min; finally, it was extended at 72 °C for 10 min. PCR products were examined by electrophoresis on 1% agarose gels. The amplified PCR products were sequenced in single direction using an ABI 3730 DNA Analyzer (Sangon, Shanghai, China) with the same primers.

### 2.4. Phylogenetic Analyses

Newly generated sequences in this study were combined with those downloaded from GenBank/UNITE for phylogenetic construction according to the previous studies [18,19,20,21,26,31,33]. Information about sequences is presented in Table 2. The sequence matrix of ITS, nrLSU, and *tef1* was separately aligned with MAFFT v7.490 and manually optimized on BioEdit v7.0.9 where necessary [42,43]. All positions with less than 50% site coverage were eliminated, i.e., fewer than 50% alignment gaps, missing data, and ambiguous bases (partial deletion option).

ITS-nrLSU-*tef1* and ITS-nrLSU datasets were analyzed using Maximum Likelihood (ML) and Bayesian Inference (BI) methods, respectively. ML analyses were implemented with RAxML-HPC v8.2.10, and 1000 rapid bootstrap replicates were performed; GTRGAMMA was set by default as the selected model [44]. For BI analyses, the most appropriate substitution models were selected by MrModeltest v2.3 under the Akaike information criterion (AIC) [45]. BI analyses were conducted using MrBayes v3.2.7 [46]. For *Hydnum*, General Time Reversible + Proportion of Invariable Sites + Gamma (GTR + I + G) for ITS and nrLSU, GTR + G for *tef1*; four Markov chains were run for 5,000,000 generations until split deviation frequency value was <0.01, sampling every 100th generation. For *Lentaria* s.l., GTR + I + G for ITS, Symmetrical Model + Proportion of Invariable Sites + Gamma (SYM + I + G) for nrLSU; four Markov chains were run for 3,000,000 generations until split deviation frequency value was <0.01, sampling every 1000th generation. Then, the first 25% of sampled trees from generations were discarded as burn-in, and Bayesian posterior probabilities (PP) were then calculated for a majority consensus tree of retained Bayesian trees. All trees were visualized in FigTree v1.4.2.

**Table 2:** Specimens and sequences used in this study.

Taxa	Voucher	Locality	GenBank/UNITE Accession Nos.			References
			ITS	nrLSU	*tef1*	
*Clavariadelphus khinganensis*	MHKMU HY Huang 368	NE China: Jilin	MT447468	—	—	[12]
*Hydnum alboaurantiacum*	TENN 073053 (T)	USA	MH379955	—	—	[23]
*H. alboaurantiacum*	TENN 073051	USA	MH379937	—	—	[23]
*H. alboluteum*	TUMH 63988 (T)	Japan	LC621802	—	LC622439	[22]
*H. alboluteum*	TUMH 63989	Japan	LC621803	—	—	[22]
*H. albomagnum*	AFTOL-ID 471	USA	DQ218305	AY700199	DQ234568	[47]
*H. albomagnum*	TENN 073062 (T)	USA	MH379943	—	—	[23]
*H. albopallidum*	TUMH 63997 (T)	Japan	LC621807	LC717904	LC622442	[22]
*H. albopallidum*	TUMH 63998	Japan	LC621808	—	LC622443	[22]
*H. berkeleyanum*	CAL 1656 (T)	India	NR_158533	NG_070500	—	[24]
*H. berkeleyanum*	HKAS77834	SW China: Yunnan	KU612525	KU612667	—	[19]
*H. berkeleyanum*	IFP 019484	China	MW980552	MW979538	—	[18]
***H. berkeleyanum***	**MHKMU YJ Pu 361**	**SW China: Yunnan**	**PQ287656**	**PQ287738**	**PQ295833**	**This study**
***H. berkeleyanum***	**MHKMU M Mu 740**	**SW China: Yunnan**	**PQ287657**	**PQ287739**	**PQ295834**	**This study**
*H. boreorepandum*	H 6003711 (T)	Finland	KX388657	—	—	[20]
*H. boreorepandum*	TUMH 64005	Japan	LC621814	LC717880	LC622449	[22,48]
*H. boreorepandum* (*H. repandum*)	HKAS54416	NE China: Jilin	KU612583	—	—	[19]
*H. brevispinum*	IFP 019464 (T)	C China: Hunan	MW980578	MW979559	—	[18]
*H. brevispinum*	IFP 019465	C China: Hunan	MW980579	MW979560	—	[18]
***H. cremeum***	**MHKMU TJ Yu 197 (T)**	**SW China: Yunnan**	**PQ287674**	**PQ287755**	**PQ295848**	**This study**
***H. cremeum***	**MHKMU WH Zhang 599**	**SW China: Yunnan**	**PQ287673**	**PQ287754**	**PQ295847**	**This study**
*H. cremeoalbum*	GDGM93011	C China: Hubei	OR947110	OR947129	—	[26]
*H. cremeoalbum*	TUMH 60740 (T)	Japan	AB906678	—	—	[25]
*H. cremeoalbum*	HKAS92345	SW China: Chongqing	KU612619	KU612676	KU612764	[19]
*H. cremeoalbum*	FHMU1631	S China: Hainan	OQ656784	OQ656792	—	[21]
*H. cremeoalbum* (*H. albomagnum*)	IFP 019480	C China: Hunan	MW980550	MW979536	—	[18]
*H. erectum*	FHMU7689 (T)	E China: Zhejiang	OR722666	OR722669	—	[21]
*H. flabellatum*	IFP 019459 (T)	NE China: Liaoning	MW980575	MW979556	—	[18]
*H. flavidocanum*	IFP 019460 (T)	SW China: Yunnan	MW980559	MW979545	MW999440	[18]
*H. flavidocanum*	IFP 019461	SW China: Yunnan	MW980560	MW979546	MW999441	[18]
***H. flavosquamosum***	**MHKMU LP Tang 3454 (T)**	**SW China: Yunnan**	**PQ287672**	**PQ287753**	**PQ295846**	**This study**
*H. ibericum*	BIO Fungi: 12330 (T)	Spain	HE611086	—	—	[28]
*H. ibericum*	MA-fungi 3457	Spain	AJ547879	—	—	[49]
*H. jussii*	H 6003709 (T)	Finland	KX388665	—	—	[20]
*H. jussii*	IFP 019485	NW China: Xinjiang	MW980553	MW979539	MW999436	[18]
*H. khanspurense*	KH-09 (LAN88021) (T)	Pakistan	OQ130694	—	—	[50]
*H. khanspurense*	KH-50 (LAN29722)	Pakistan	OQ130695	—	—	[50]
*H. longibasidium*	IFP 019462 (T)	C China: Hunan	MW980556	MW979541	MW999438	[18]
*H. longibasidium*	IFP 019463	C China: Hunan	MW980555	MW979542	MW999439	[18]
*H. longipes*	GDGM82458 (T)	SW China: Yunnan	OR947121	—	—	[26]
*H. melitosarx*	GDGM84518	SW China: Sichuan	OR947117	OR947136	—	[26]
*H. melitosarx*	H7043937 (T)	USA	KX388683	—	—	[20]
*H. melitosarx* (*H. rufescens*)	HKAS92338	NE China: Jilin	KU612538	KU612659	KU612784	[19]
*H. microcarpum*	GDGM87902 (T)	S China: Guangdong	OR947116	OR947134	—	[26]
*H. microcarpum*	GDGM87902-1	S China: Guangdong	OR947115	—	—	[26]
*H. minum*	TUMH 60737 (T)	Japan	AB906675	—	—	[25]
*H. minum*	IFP 019482	C China: Hunan	MW980557	MW979543	—	[18]
***H. minum***	**MHKMU YR Li 004**	**SW China: Yunnan**	**PQ287659**	**PQ287741**	**—**	**This study**
***H. minum***	**MHKMU SD Yang 378**	**SW China: Yunnan**	**PQ287658**	**PQ287740**	**—**	**This study**
*H. minum*	FHMU2408	S China: Hainan	OQ656785	OQ656793	—	[21]
*H. orientalbidum*	GDGM93480	SW China: Chongqing	OR947108	OR947127	—	[26]
*H. orientalbidum*	GDGM91301	E China: Zhejiang	OR947111	OR947130	—	[26]
*H. orientalbidum*	TUMH 62998 (T)	Japan	LC377875	LC717908	LC622478	[48,51]
*H. orientalbidum*	FHMU6327	S China: Hainan	OQ656787	OQ656794	—	[21]
*H. pallidocroceum*	IFP 019466 (T)	NW China: Xinjiang	MW980568	MW979554	MW999449	[18]
*H. pallidocroceum*	IFP 019467	NW China: Xinjiang	MW980569	MW979555	MW999450	[18]
*H. pallidomarginatum*	IFP 019468 (T)	SW China: Yunnan	MW980566	MW979552	MW999447	[18]
***H. pallidomarginatum***	**MHKMU HY Huang 873**	**SW China: Yunnan**	**PQ287660**	**PQ287742**	**PQ295835**	**This study**
***H. pallidomarginatum***	**MHKMU LP Tang 3319**	**SW China: Yunnan**	**PQ287661**	**PQ287743**	**PQ295836**	**This study**
***H. pallidomarginatum***	**MHKMU LP Tang 3453**	**SW China: Yunnan**	**PQ287662**	**PQ287744**	**PQ295837**	**This study**
***H. pallidomarginatum***	**MHKMU SD Yang 557**	**SW China: Yunnan**	**PQ287663**	**PQ287745**	**PQ295838**	**This study**
***H. pallidomarginatum***	**MHKMU M Mu 791**	**SW China: Yunnan**	**PQ287664**	**PQ287746**	**PQ295839**	**This study**
*H. pallidomarginatum* (*H. vesterholtii*)	HKAS92344	NE China: Heilongjiang	KU612556	KU612649	KU612788	[19]
*H. pallidomarginatum* (*H. vesterholtii*)	HKAS56213	SW China: Yunnan	KU612554	—	—	[19]
*H. pinicola*	GDGM93020	C China: Hubei	OR947109	OR947128	—	[26]
*H. pinicola*	GDGM83047	SW China: Yunnan	OR947119	OR947137	—	[26]
*H. pinicola*	TUMH 64004 (T)	Japan	LC621813	—	LC622448	[22]
*H. repandum*	031209A	Slovenia	KU612574	KU612655	KU612770	[19]
*H. repandum*	H 6003710 (T)	Finland	NR_164553	—	—	[20]
***H. roseoalbum***	**MHKMU WH Zhang 606 (T)**	**SW China: Yunnan**	**PQ287669**	**PQ287751**	**PQ295844**	**This study**
***H. roseoalbum***	**MHKMU WH Zhang 606-1**	**SW China: Yunnan**	**PQ287670**	**PQ287752**	**PQ295845**	**This study**
***H. roseotangerinum***	**MHKMU LP Tang 3458 (T)**	**SW China: Yunnan**	**PQ287675**	**PQ287756**	**PQ295849**	**This study**
***H. roseotangerinum***	**MHKMU LP Tang 3458-1**	**SW China: Yunnan**	**PQ287676**	**PQ287757**	**PQ295850**	**This study**
*H. sinorepandum*	GDGM82445 (T)	SW China: Yunnan	OR947122	OR947139	—	[26]
*H. sinorepandum*	GDGM82382	SW China: Yunnan	OR947124	OR947141	—	[26]
*Hydnum* sp.	HMJAU60215	China	OM341393	—	—	GenBank
*Hydnum* sp.	HMJAU60216	China	OM341394	—	—	GenBank
*Hydnum* sp. 1 (*H. vesterholtii*)	HKAS92342	SW China: Yunnan	KU612564	KU612646	KU612786	[19]
*Hydnum* sp. 1 (*H. vesterholtii*)	HKAS77884	C China: Hubei	KU612565	KU612645	KU612787	[19]
*Hydnum* sp. 2 (*H. vesterholtii*)	HKAS92341	NW China: Shaanxi	KU612562	KU612647	KU612790	[19]
***Hydnum* sp. 3**	**MHKMU LP Tang 2886**	**NE China: Jilin**	**PQ287671**	**—**	**—**	**This study**
*Hydnum* sp. 3 (*H. vesterholtii*)	HKAS92343	SW China: Sichuan	KU612563	KU612648	—	[19]
*H. sphaericum*	IFP 019470 (T)	C China: Hunan	MW980563	MW979549	MW999444	[18]
***H. sphaericum***	**MHKMU X Na 149**	**SW China: Yunnan**	**PQ287665**	**PQ287747**	**PQ295840**	**This study**
*H. subalpinum*	TUMH 64013	Japan	LC717913	LC717888	LC717874	[48]
*H. subalpinum*	TUMH 64016 (T)	Japan	LC621871	LC717891	LC622497	[22,48]
*H. subtilior*	TENN 073050	USA	MH379918	—	—	[23]
*H. subtilior*	TENN 073034 (T)	USA	NR_164029	—	—	[23]
*H. tangerinum*	IFP 019473 (T)	C China: Hunan	MW980580	MW979561	—	[18]
*H. tangerinum*	IFP 019474	C China: Hunan	MW980581	MW979562	—	[18]
*H. tenuistipitum*	IFP 019476 (T)	C China: Hunan	MW980576	MW979557	—	[18]
*H. tenuistipitum*	FHMU7644	C China: Hunan	OQ913759	OQ913756	—	[21]
***H. tenuistipitum***	**MHKMU HY Huang 151**	**SW China: Yunnan**	**PQ287666**	**PQ287748**	**PQ295841**	**This study**
***H. tenuistipitum***	**MHKMU LP Tang 2116**	**SW China: Yunnan**	**PQ287667**	**PQ287749**	**PQ295842**	**This study**
***H. tenuistipitum***	**MHKMU J Zhao 225**	**SW China: Yunnan**	**PQ287668**	**PQ287750**	**PQ295843**	**This study**
*H. tomaense*	TUMH 64086 (T)	Japan	LC621885	LC717907	LC622509	[22,48]
*H. tomaense*	TUMH 64085	Japan	LC621884	—	LC622508	[22]
*H. treui*	TU110403 (T)	Papua New Guinea	UDB01304 *	—	—	[20,52]
*H. treui*	FHMU7690	S China: Hainan	OR722667	OR722670	—	[21]
*H. umbilicatum*	CORT: 012241 (T)	USA	MH379890	—	—	[23]
*H. umbilicatum*	TUMH 64093	Japan	LC621893	—	—	[22]
*H. umbilicatum* (*H. ellipsosporum*)	HMJAU5985	NE China: Jilin	KU612602	—	—	[19]
*H. ventricosum*	IFP 019478 (T)	NE China: Liaoning	MW980561	MW979547	MW999442	[18]
*H. ventricosum*	IFP 019479	NE China: Liaoning	MW980562	MW979548	—	[18]
*H. vesterholtii*	BIO Fungi: 12904 (T)	France	HE611087	—	—	[28]
*H. zongolicense*	MEXU 26248 (T)	Mexico	KC152121	—	—	[20]
*Lentaria albovinacea*	FO 46869	Unknown	DQ071734	—	—	[53]
*L. bambusina*	MHHNU 6794 (T)	C China: Hunan	KU870448	—	—	[31]
*L. bambusina*	MHHNU 7302	C China: Hunan	KU324496	—	—	[31]
***L. bambusina***	**MHKMU C Xu 071**	**E China: Anhui**	**PQ287679**	**PQ287760**	**—**	**This study**
*L. byssiseda*	TENN61159-C2	USA: Tennessee	FJ596785	—	—	[54]
*L. byssiseda*	TENN61159-C3	USA: Tennessee	FJ596786	—	—	[54]
*L. gossypina*	FCME 27625	Mexico: Campeche	MK253199	MK253219	—	[33]
*L. gossypina*	FCME 27624	Mexico: Campeche	MK253198	MK253218	—	[33]
*L. micheneri*	RRD6	USA: Tennessee	MF773634	—	—	GenBank
*L. micheneri*	iNaturalis 178123461	USA: Tennessee	PP526130	—	—	GenBank
*L. patouillardii*	MHHNU 7829	NW China: Xinjiang	KU324498	—	—	[31]
*L. patouillardii*	HMJAU 26892	NW China: Inner Mongolia	KU870449	—	—	[31]
*L. patouillardii*	MA-Fungi 48032	Spain	AJ292290	—	—	GenBank
***L. subalpina***	**MHKMU TJ Yu 206 (T)**	**SW China: Yunnan**	**PQ287677**	**PQ287758**	**—**	**This study**
***L. subalpina***	**MHKMU X Xia 129**	**SW China: Yunnan**	**PQ287678**	**PQ287759**	**—**	**This study**
*L. surculus*	MHHNU 8721	SW China: Yunnan	KU870450	—	—	[31]
*L. surculus*	FHMU 880	S China: Guangdong	KU870451	—	—	[31]
***L. surculus***	**MHKMU LJ Su 430**	**S China: Hainan**	**PQ287680**	**PQ287761**	**—**	**This study**
***L. surculus***	**MHKMU LJ Su 431**	**S China: Hainan**	**PQ287681**	**PQ287762**	**—**	**This study**
*L. uncispora*	MHHNU 7707 (T)	SW China: Sichuan	KU324497	—	—	[31]
*L. variabilis*	FCME 21524	Mexico: Campeche	MK253189	MK253215	—	[33]
*L. variabilis*	FCME 19864	Mexico: Campeche	MK253185	MK253206	—	[33]
*Sistotrema muscicola*	KHL 11721	Finland	AJ606040	AJ606040	—	[55]
*S. muscicola*	taxon: 154757	Finland	AJ606041	AJ606041	—	[55]

Newly generated sequences are highlighted in bold. ‘T’ refers to type specimens; * refers to sequence retrieved from UNITE; wrong names in GenBank are put in brackets, e.g., *H. pallidomarginatum* (*H. vesterholtii*); C = Central, E = Eastern, NE = Northeastern, NW = Northwestern, S = Southern, SW = Southwestern.

## 3. Results

### 3.1. Molecular Phylogeny

#### 3.1.1. Genus Hydnum

**Figure 2:**
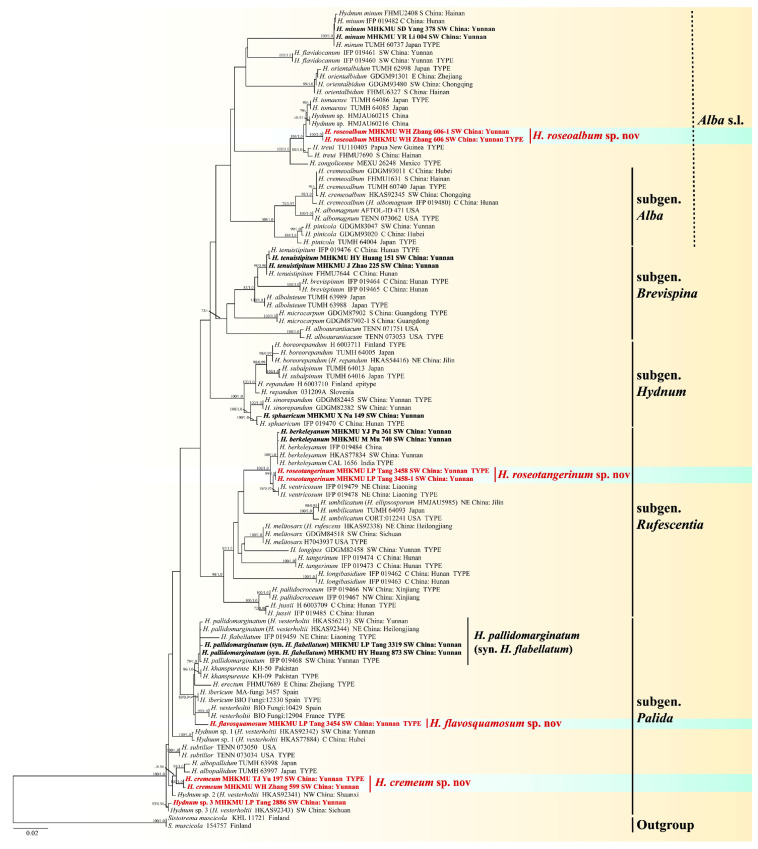
Phylogenetic tree of *Hydnum* species based on ITS-nrLSU-*tef1* sequences. Bootstrap values ≥ 70% and Bayesian posterior probabilities values ≥ 0.95 are shown on branches (newly generated sequences in bold and new species in red).

In the ITS-nrLSU-*tef1* dataset, 105 ITS, 72 nrLSU, and 46 *tef1* sequences were included, including 59 newly generated and 164 retrieved ones from GenBank/UNITE, respectively. *Sistotrema muscicola* (Pers.) S. Lundell was selected as an outgroup based on recent studies [18,21]. The combined dataset includes 104 taxa with 2357 nucleotide sites, and the alignment is available at TreeBase (ID: 31750). The topology of the phylogenetic trees obtained from the combined and the single-locus matrices were essentially the same, and we only show the results of the combined matrix here; the results of the single-locus are detailed in the Supplementary Materials. The ITS-nrLSU-*tef1* phylogenetic tree is shown in Figure 2.

The topologies obtained from the ITS-nrLSU-*tef1* datasets in our study were similar to the phylograms from previous studies [18,21,22,26]. Newly generated sequences were classified into ten clades with high support values (BS/BPP ≥ 90/0.95), including four new species. Among these taxa, *H. cremeum* sp. nov from China formed a distinct lineage (BS/BPP = 100/1) in the subgen. *Pallida*, and it was a sister species of *H. albopallidum* R. Sugaw. & N. Endo from Japan (BS/BPP = 60/0.96); *H. flavosquamosum* sp. nov formed a distinct lineage in the subgen. *Pallida*, but there was no resolution among the closely related phylogenetic species; *H. roseoalbum* sp. nov was grouped in the subgen. *Alba*. s. lato (BS/BPP = 100/1); *Hydnum roseotangerinum* sp. nov from China in the subgen. *Rufescentia* was closely related to two Asian species, *H. berkeleyanum* K. Das, Hembrom, A. Baghela & Vizzini and *H. ventricosum* T. Cao & H. S. Yuan.

#### 3.1.2. Genus *Lentaria* s.l.

**Figure 3:**
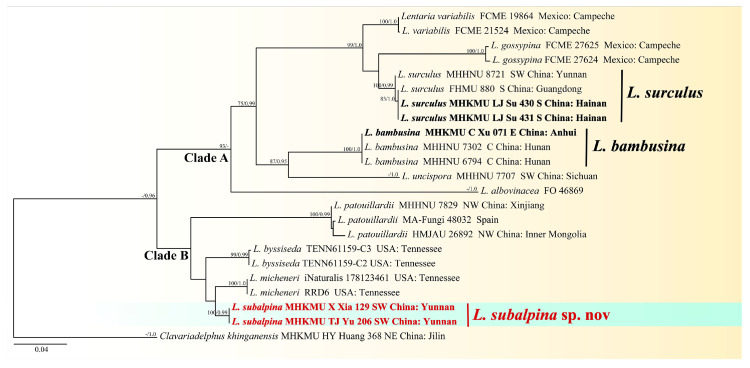
Phylogenetic tree of *Lentaria* s.l. species based on ITS-nrLSU sequences. Bootstrap values ≥ 70% and Bayesian posterior probabilities values ≥ 0.95 are shown on branches (newly generated sequences in bold and new species in red).

In the ITS-nrLSU dataset, 34 sequences were included, including ten newly generated and 24 retrieved ones from GenBank. *Clavariadelphus khinganensis* J. Zhao, L.P. Tang & P. Zhang was used as the outgroup based on recent studies [12,31]. The combined dataset includes 23 taxa with 1602 nucleotide sites, and the alignment is available at TreeBase (ID: 31752). The topology of the phylogenetic trees obtained from the combined and the single-locus matrix were essentially the same, and we only show the results of the combined matrix here; the results of the single-locus are detailed in the Supplementary Materials. The phylogenetic tree is shown in Figure 3.

Our topologies obtained from the ITS-nrLSU dataset showed that the genus *Lentaria* s.l. was a polyphyletic group consisting of two clades. This result was similar to the phylograms in previous studies [31,33]. *Lentaria subalpina* sp. nov was closely related to *L. byssiseda* Corner and *L. micheneri* (Berk. & M.A. Curtis) Corner, while this new species had no identified sister species in this study.

### 3.2. Taxonomy

**Figure 4:**
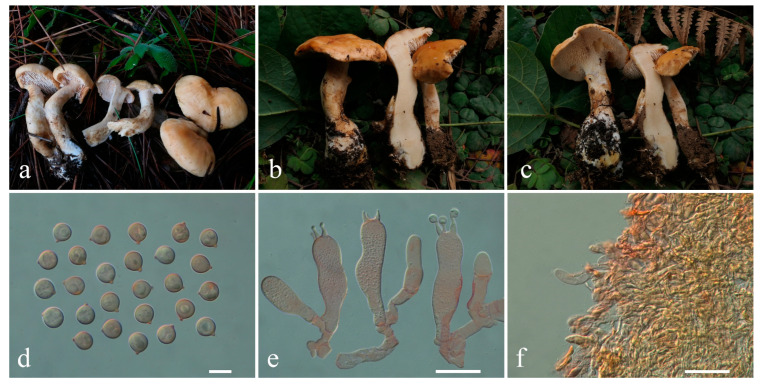
Macroscopic and microscopic features of *Hydnum berkeleyanum*. (**a**–**c**) Basidiomata, (**a**) from MHKMU YJ Pu 361; (**b**,**c**) from MHKMU M Mu 740; (**d**) Basidiospores; (**e**) Basidia and Basidioles; (**f**) Pileipellis. Bars (**d**) = 10 μm; (**e**) = 20 μm; (**f**) = 50 μm.

***Hydnum berkeleyanum*** K. Das, Hembrom, A. Baghela & Vizzini. Cryptogam. Mycol. 39(2): 212, 2018 (Figure 4a–f)

Chinese name—贝氏齿菌 [29]

MycoBank—MB 824501

Description—*Basidiomata* small-sized. *Pileus* 4–5 cm in diam., plano-convex to plane, irregular round, sometimes with irregular lobes or umbilicate in the center; surface dry, irregularly bumpy, non-staining, azonate, pale orange when young, becoming yellowish brown to orange-brown when mature, paler towards margin; margin entire, incurved. *Context* 0.5–0.7 cm thick in pileal center, fleshy, and warm cream to yellowish white. *Stipe* 6 cm long, 0.9–1.6 cm in diam., central to eccentric, cylindrical, white to pale orange-brown, solid interior, the base often inflated. *Spines* 0.4–0.5 cm long (with some shorter spines on stipe), adnexed to decurrent, not flattened, with an acute (rarely subacute) apex, crowded, creamy, gradually more yellowish when drying. *Odor* and *Taste* not recorded.

*Basidiospores* [40/2/2] 8.1–9.0 (–9.5) × 8.1–9.0 (–9.5) µm, Q = 1.00–1.06 (–1.11), Qm = 1.02 ± 0.03, globose, sometimes subglobose, thin-walled, inamyloid. *Basidia* 31–64 (–82) × 9.5–14 µm, subcylindric to clavate, 2- to 4-spored, clamped; sterigmata 2.5–8.5 µm long. *Basidioles* numerous, subcylindrical or subclavate, smaller than basidia. *Cystidia* absent. *Spine tip* sterile, composed of interwoven hyphae (3.5–11 µm wide), septate, clamped, apex subclavate to clavate, becoming parallel towards the apex. *Hymenophoral trama* composed of hyphae (2.5–6 µm wide), clamped, in subparallel to interwoven pattern. *Pileipellis* composed of densely interwoven, suberect hyphae (5–14 µm wide), cylindrical, thin-walled, clamped, the terminal cell rounded at the apex, with pale yellow intracellular pigment. *Clamp connections:* present.

Habitat—Solitary or concrescent on the ground of temperate to subalpine mixed forests dominated by Ericaceae, Fagaceae, and Pinaceae.

Specimens examined—CHINA. Yunnan Province, Shuhe Ancient Town (束河古镇), N 26°58′26″, E 100°10′47″, elev. 3180 m, 22 August 2020, *Mu Man 740* (MHKMU M Mu 740); about 3 km from Alpine Botanical Garden (高山植物园), N 26°58′26″, E 100°10′47″, elev. 3180 m, 22 August 2020, on the ground of subalpine mixed forests dominated by Ericaceae, Fagaceae, and Pinaceae, *Pu Yunju 361* (MHKMU YJ Pu 361).

Known distribution—Asia: Yunnan Province, China, elev. above 3000 m [18,19,24,26]; India (type location), elev. 1800–2200 m [24]; and Pakistan, elev. 2200 m–2500 m [50].

Notes—*Hydnum berkeleyanum* is characterized by its pale orange to orange-brown colored pileus, 2- to 4-spored basidia, and globose basidiospores. It was described from India, with reports also found in Pakistan and China [18,24,26,50]. We examined collections from Yunnan and several DNA sequences from China labeled as *H. berkeleyanum* in the database. No major differences were found between our specimen and the type, except for the size of basidiomata. Additionally, Qin et al. [21] mistakenly recorded the collecting site of HKAS77834 as Hunan Province, China. After reviewing the literature, we determined that this specimen was taken from Yunnan Province rather than Hunan [19]. Thus, the known distribution of *H. berkeleyanum* in China is limited to the southwest rather than the central regions.

**Figure 5:**
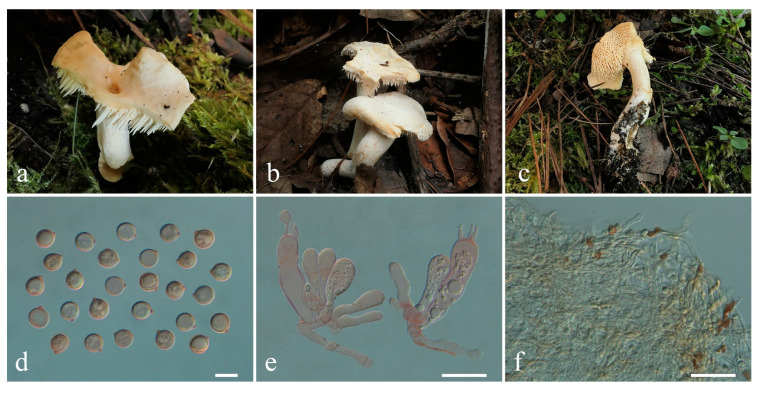
Macroscopic and microscopic features of *Hydnum cremeum*. (**a**–**c**) Basidiomata, (**a**,**c**) from MHKMU TJ Yu 197, Holotype; (**b**) from MHKMU WH Zhang 599; (**d**) Basidiospores; (**e**) Basidia and Basidioles; (**f**) Pileipellis. Bars (**d**) = 10 μm; (**e**) = 20 μm; (**f**) = 50 μm.

***Hydnum cremeum*** L.P. Tang, L.J. Su & T.J. Yu sp. nov. (Figure 5a–f)

Chinese name—奶油齿菌

MycoBank—MB 855827

Etymology—Latin ‘*cremeum*’ = cream, refers to the color of basidiomata.

Diagnosis—Distinct from other species within *Hydnum* mainly by its small basidiomata with a warm cream to yellowish white pileus, globose to subglobose basidiospores, and occurrence in subalpine mixed forests.

Holotype—CHINA. Yunnan Province, Ninglang County (宁蒗县), Gewa Village (格瓦村), N 27°42′34″, E 100°31′′30″, elev. 3116 m, 2 October 2023, on the ground of subalpine mixed forests dominated by *Pinus yunnanensis* Franch., *P. densata* Mast., *Quercus* L., and *Rhododendron decorum* Franch., *Yu Taijie 197* (MHKMU TJ Yu 197). GenBank accession numbers: ITS = PQ287674, nrLSU = PQ287755, *tef1* = PQ295848.

Description—*Basidiomata* very small- to small-sized. *Pileus* 2.5–3.7 cm in diam., plano-convex to plane, irregular round, central sometimes depress, shallow infundibuliform when old; surface dry, irregularly bumpy, non-staining, azonate, warm cream (3A1) to yellowish white (3–4A2), with light yellow (5A5) to light grayish-orange (5B4–5) patches; margin entire, incurved. *Context* 0.2–0.3 cm thick in pileal center, fleshy, warm cream, slightly becoming yellowish white to pale orange on exposure. *Stipe* 3–6 cm long, 0.5–1 cm in diam., central, eccentric to lateral, cylindrical, concolorous with the pileus, solid interior. *Spines* 0.4–0.5 cm long (with some shorter spines on stipe), adnexed to slightly decurrent, not flattened, with an acute (rarely subacute) apex, crowded, cream, gradually more yellow when drying. *Odor* and *Taste* not recorded.

*Basidiospores* [80/3/2] 8.1–9.5 × 7.1–9.0 (–9.5) µm, Q = 1.00–1.11 (–1.13), Qm = 1.05 ± 0.04, globose to subglobose, thin-walled, inamyloid. *Basidia* 40–57 × 9.5–13 µm, subcylindric to clavate, 2- to 4-spored, clamped; sterigmata 5–9.5 µm long. *Basidioles* numerous, subcylindrical or subclavate, smaller than basidia. *Cystidia* absent. *Spine tip* sterile, composed of interwoven hyphae (4–11.5 µm wide), septate, clamped, apex subclavate to clavate, becoming parallel towards the apex. *Hymenophoral trama* composed of hyphae (2.5–6 µm wide), clamped, in subparallel to interwoven pattern. *Pileipellis* composed of densely interwoven, subparallel to suberect hyphae (3.5–9.5 µm wide), cylindrical, thin-walled, clamped, the terminal cell rounded at the apex, with pale yellow intracellular pigment. *Clamp connections* present.

Habitat—Solitary or concrescent on the ground of subtropical to subalpine mixed forests dominated by Ericaceae, Fagaceae, and Pinaceae.

Additional specimens examined—CHINA. Yunnan Province, Jianchuan County (剑川县), Shibaoshan Scenic Spot (石宝山景区), N 26°23′41″, E 99°50′10″, elev. 2500 m, 5 October 2020, on the ground of subtropical broad-leaved forests dominated by Fagaceae and Ericaceae, *Zhang Wenhao 599* (MHKMU WH Zhang 599).

Known distribution—China: Yunnan Province, elev. above 2500 m.

Notes—*Hydnum cremeum* is easily distinguished by its small-sized, cream basidiomata, with light yellow patches, sometimes a shallow infundibuliform pileus, 2–4-spored basidia, and globose to subglobose basidiospores. In China, *H. cremeum* is often misidentified with *H. orientalbidum* R. Sugaw. & N. Endo, *H. pallidomarginatum* T. Cao & H.S. Yuan, and *H. pinicola* R. Sugaw. & N. Endo. However, *H. orientalbidum* has slightly larger basidiomata (pileus up to 5.5 cm in diam.), 3–5 (7)-spored basidia, and smaller basidiospores (4.5–6 × 4–5 µm) [22]; *H. pallidomarginatum* has a zonate, orange white to pale orange pileus, concolorous spines, 2–4-spored basidia, and longer basidiospores (8.2–9.8 × 6.5–7.8 μm) [18]; *H. pinicola* has pale orange spines, 4–8-spored basidia, and smaller basidiospores (4.5–5.5 × 4–5 µm) [22,26]. Furthermore, *H. sinorepandum* Ming Zhang & C.Q. Wang and *H. subalpinum* R. Sugaw. & N. Endo are also distributed in the subalpine regions of Asia and have a creamy to yellowish white pileus. In contrast, *H. sinorepandum* has large basidiomata (pileus up to 12 cm in diam) with a light orange tinge, 4–8-spored basidia, and broadly ellipsoid basidiospores [26]; *H. subalpinum* has robust basidiomata, 3–5-spored basidia, relatively narrower basidiospores (Q = 1.03–1.23), and occurrence in coniferous forests in Japan [22,26,48]. Additionally, *H. cremeum* is sister to *H. albopallidum*, but the latter has an umbilicate pileus, (2) 3–4-spored basidia, narrower basidiospores (8–9.5 × 7–8 µm), and is only found in Japan to date [22].

**Figure 6:**
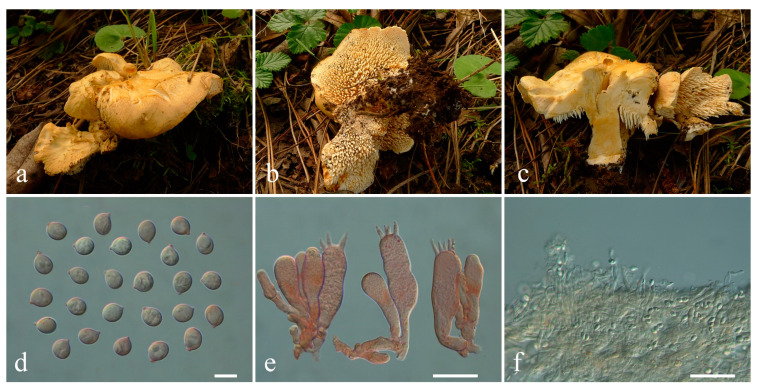
Macroscopic and microscopic features of *Hydnum flavosquamosum* from MHKMU LP Tang 3454 (Holotype). (**a**–**c**) Basidiomata; (**d**) Basidiospores; (**e**) Basidia and Basidioles; (**f**) Pileipellis. Bars (**d**) = 10 μm; (**e**) = 20 μm; (**f**) = 50 μm.

***Hydnum flavosquamosum*** L.P. Tang & L.J. Su sp. nov. (Figure 6a–f)

Chinese name—黄鳞齿菌

MycoBank—MB 855834

Etymology—Latin ‘*flavo*’ = yellow, ‘*squamosum*’ = scaly, refers to the pileus covering with yellowish brown scales.

Diagnosis—Distinct from other species within *Hydnum* mainly by its light yellow to light brownish-orange pileus with slightly dark scales, context becoming pale brownish on exposure, adnexed, spines, subglobose to broadly ellipsoid (Q = 1.05–1.23) basidiospores, and erectly arranged hyphae in pileipellis.

Holotype—CHINA. Yunnan Province, Yi Family Village (彝家村), N 27°23′14″, E 100°06′ 31″, elev. 3170 m, 26 August 2020, on the ground of subalpine mixed forests dominated by *Picea likiangensis* (Franch.) E. Pritz., *Pinus armandi* Franch., *P. densata*, *Quercus guyavifolia* H. Lév., and *Rhododendron decorum*, *Tang Liping 3454* (MHKMU LP Tang 3454). GenBank accession numbers: ITS = PQ287672, nrLSU = PQ287753, *tef1* = PQ295846.

Description—*Basidiomata* medium-sized. *Pileus* 5 cm in diam., plano-convex to plane, easily cracked; surface dry, azonate, light yellow (4A4–5) to light brownish-orange (5B4–5), covered with light orange (5A5), brownish yellow (5C7), grayish orange (5B6) to light brown (5D8) scales; margin split, incurved. *Context* 0.5 cm thick in pileal center, fleshy, cream to yellowish white, slightly becoming pale brownish on exposure, eventually turns light reddish-brown (6C6). *Stipe* 2 cm long, 0.9 cm in diam., eccentric to lateral, cylindrical, cream to light yellow, solid interior. *Spines* 0.5 cm long, adnexed, non-decurrent, not flattened, with an acute apex, crowded, light yellow to light brownish-orange, gradually more yellow when drying. *Odor* and *Taste* not recorded.

*Basidiospores* [40/1/1] 8.6–9.5 (–10.0) × 7.6–8.6 (–9.0) µm, Q = (1.00–) 1.05–1.23 (–1.25), Qm = 1.12 ± 0.06, subglobose to broadly ellipsoid, rarely globose, thin-walled, inamyloid. *Basidia* 37–58 × 9.5–13 µm, subcylindric to clavate, 2- to 5-spored, clamped; sterigmata 4–7.5 µm long. *Basidioles* numerous, subcylindrical or subclavate, smaller than basidia. *Cystidia* absent. *Spine tip* sterile, composed of interwoven hyphae (5–10 µm wide), septate, clamped, apex subclavate to clavate, becoming parallel towards the apex. *Hymenophoral trama* composed of hyphae (2–6 µm wide), clamped, in subparallel to interwoven pattern. *Pileipellis* composed of densely interwoven, erect hyphae (3–7 µm wide), cylindrical to subfusiform, thin-walled, clamped, the terminal cell rounded at the apex, with pale yellow intracellular pigment. *Clamp connections* present.

Habitat—Solitary on the ground of subalpine mixed forests dominated by Ericaceae, Fagaceae, and Pinaceae.

Known distribution—China: Yunnan Province, elev. about 3100 m.

Notes—*Hydnum flavosquamosum* has a yellow to light brownish-colored pileus covered with slightly dark scales, non-decurrent spines, 2–5-spored basidia, and subglobose to broadly ellipsoid basidiospores. It is similar to *H. berkeleyanum*, *H. rufescens* Pers., and *H. subrufescens* Niskanen & Liimat, but they can be distinguished by their darker pileus. Additionally, *H. berkeleyanum* has larger and more robust basidiomata (pileus up to 8 cm in diam.), a brown pileal center, longer spines (9 mm), 2–4-spored basidia, and distributing in temperate to subtropical regions [24,29]; *H. rufescens* has a deep reddish-orange to brownish-orange pileus, without scales, 3–5-spored basidia, smaller basidiospores (7.0–8.5 × 6.0–7.2 μm), and grows in European mixed forests [20]; *H. subrufescens* has a brownish ochraceous and non-squamous pileus, 3–4-spored basidia, and smaller basidiospores (7.4–8.8 × 6.4–7.8 μm), with a distribution limited to eastern North America [20]. *Hydnum erectum* N.K. Zeng, H.Z. Qin, W.F. Lin & L.G. Hu also has erectly arranged hyphae in the pileipellis, but it has a creamy pileus, shorter spines (1–2 mm long), 2–4-spored basidia, and small basidiospores (6.5–8 × 5.5–7.5 μm) [21]. In addition, *H. flavosquamosum* is closely related to species in the subgen. *Pallida* phylogenetically. But species in the subgen. *Pallida* always have paler basidiomata and mostly ovoid to broadly ellipsoid basidiospores (Q > 1.25) [18,20,23].

**Figure 7:**
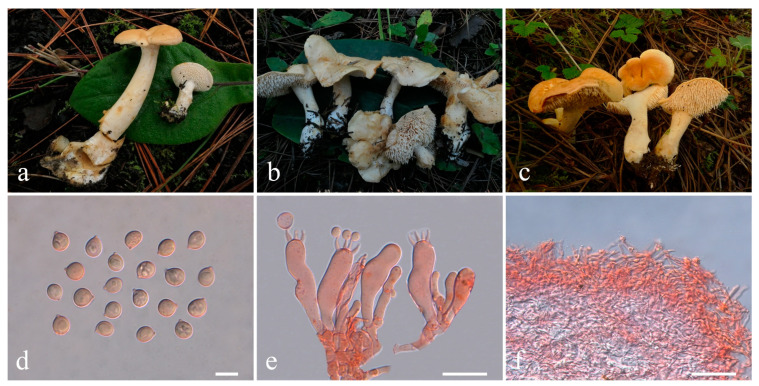
Macroscopic and microscopic features of *Hydnum pallidomarginatum*. (**a**–**c**) Basidiomata, (**a**) from MHKMU HY Huang 873, (**b**) from MHKMU M Mu 791, (**c**) from MHKMU LP Tang 3453; (**d**) Basidiospores; (**e**) Basidia and Basidioles; (**f**) Pileipellis. Bars (**d**) = 10 μm; (**e**) = 20 μm; (**f**) = 50 μm.

***Hydnum pallidomarginatum*** T. Cao & H.S. Yuan, Stud. Mycol. 99: 100121, 2021 (Figure 7a–f)

Synonym—*Hydnum flabellatum* T. Cao & H.S. Yuan, Stud. Mycol. 99: 100121, 2021, syn. nov.

Chinese name—淡缘齿菌

MycoBank—MB 839419

Description—Morphological characteristics are described in detail by Cao et al. [18].

Specimens examined—CHINA. Yunnan Province, Shizong County (师宗县), N 24°39′27′′, E 104°10′17′′, elev. 2205 m, 24 August 2018, on the ground of subtropical broad-leaved forests dominated by Ericaceae, Fagaceae, and Pinaceae, Yang Shuda 557 (MHKMU SD Yang 557); Yulong County (玉龙县), N 26°46′11′′, E 100°02′51′′, elev. 2890 m, 21 August 2020, on the ground of subalpine mixed forests dominated by *Pinus yunnanensis*, *Quercus semecarpifolia* Sm., and *Rhododendron* spp., *Tang Liping 3319* (MHKMU LP Tang 3319); Mianshaba (棉沙坝), N 27°21′16″, E 100°09′ 37″, elev. 2731 m, 24 August 2020, on the ground of subalpine mixed forests dominated by *P. yunnanensis*, *Quercus* spp., and *Rhododendron* spp., *Huang Hongyan 873* (MHKMU HY Huang 873); Yi Family Village (彝家村), N 27°23′14″, E 100°06′ 31″, elev. 3170 m, 26 August 2020, on the ground of subalpine mixed forests dominated by *Picea likiangensis*, *Pinus armandi*, *P. densata*, *P. yunnanensis*, *Q. guyavifolia*, and *R. decorum*, *Tang Liping 3453* (MHKMU LP Tang 3453); in the same location, N 27°23′14″, E 100°06′19″, elev. 3240 m, 26 August 2020, on the ground of subalpine mixed forests dominated by *P. armandi*, *Quercus* spp., and *Rhododendron* spp., *Mu Man 791* (MHKMU M Mu 791)

Known distribution—China: Heilongjiang (this study), elev. unknown, Liaoning [18], elev. unknown, and Yunnan Province [18], elev. 2200–3300 m (this study).

Notes—In 2021, *H. flabellatum* and *H. pallidomarginatum* were described from China [18]. The latter has a pale orange pileus with a paler margin, sometimes infundibuliform, decurrent spines, broadly ellipsoid basidiospores, and a distribution from temperate to subtropical [18]. In our phylogenetic analyses, *H. flabellatum* is grouped with *H. pallidomarginatum* in the subgen. *Pallida*, but there were no significant genetic differences. We rechecked the holotype’s ITS sequence of *H. flabellatum*, and it differed from *H. pallidomarginatum* by fourteen nucleotide sites. However, nine of them appeared at non-variable sites in the genus *Hydnum*. It is clear that the nucleotide site differences of this taxon arose from the poor-quality sequence. Additionally, both taxa had overlapping distributions in temperate forests of northeastern China. Moreover, the author also mentioned that the two taxa had a quite high similarity in their macro- and micro-morphology with minor differences in *H. flabellatum* having a scabrous pileus, longer stipes, 2–5-spored basidia, and the dimensions of the hyphae [18]. Therefore, we proposed that *H. flabellatum* and *H. pallidomarginatum* were con-species, and *H. flabellatum* should be a synonym of *H. pallidomarginatum*.

**Figure 8:**
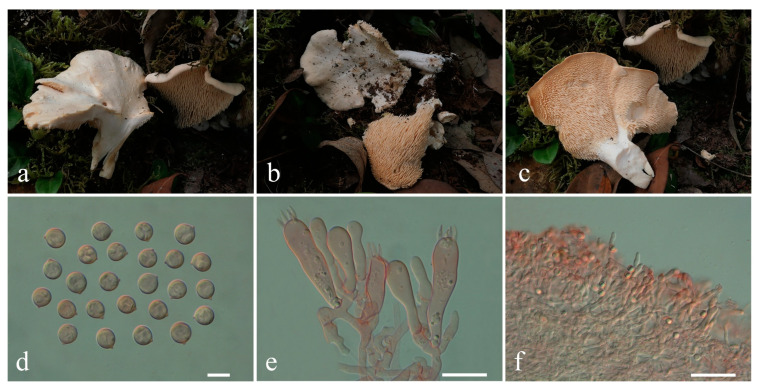
Macroscopic and microscopic features of *Hydnum roseoalbum* from MHKMU WH Zhang 606 (Holotype). (**a**–**c**) Basidiomata; (**d**) Basidiospores; (**e**) Basidia; (**f**) Pileipellis. Bars (**d**) = 10 μm; (**e**) = 20 μm; (**f**) = 50 μm.

***Hydnum roseoalbum*** L.P. Tang, L.J. Su & W.H. Zhang sp. nov. (Figure 8a–f)

Chinese name—粉白齿菌

MycoBank—MB 855835

Etymology—Latin ‘*roseo*’ = pink, ‘*album*’ = white, refers to the color of its spines.

Diagnosis—Distinct from other species within *Hydnum* by its creamy to whitish pileus, pale pink spines, five-spored basidia, globose basidiospores, erectly arranged hyphae in pileipellis, and occurrence in subalpine broad-leaved forests.

Holotype—CHINA. Yunnan Province, Jianchuan County (剑川县), N 26°17′52″, E 99°46′9″, elev. 2954 m, 6 October 2020, on the ground of subalpine broad-leaved forests dominated by Ericaceae and Fagaceae, *Zhang Wenhao 606* (MHKMU WH Zhang 606). GenBank accession numbers: ITS = PQ287669, nrLSU = PQ287751, *tef1* = PQ295844.

Description—*Basidiomata* medium-sized. *Pileus* 6.5 cm in diam., plano-convex; surface dry, sometimes with spines, azonate, cream to whitish; margin entire, incurved. *Context* 0.5 cm thick in pileal center, fleshy, white. *Stipe* 7.5 cm long, 1.4 cm in diam., eccentric, cylindrical, white to warm cream, solid interior. *Spines* 0.5–0.7 cm long (with some shorter spines on stipe), adnexed to decurrent, not flattened, with an acute (rarely subacute) apex, slightly distant, pale pink to pale orange, gradually more yellow when drying. *Odor* and *Taste* not recorded.

*Basidiospores* [40/2/2] 8.1–9.0 (–9.5) × 8.1–9.0 µm, Q = 1.00–1.06 (–1.11), Qm = 1.02 ± 0.03, globose, sometimes subglobose, thin-walled, inamyloid. *Basidia* 38–72 × 10.5–12 µm, subcylindric to clavate, 2- to 5-spored, clamped; sterigmata 4.5–9.5 µm long. *Basidioles* numerous, subcylindrical or subclavate, smaller than basidia. *Cystidia* absent. *Spine tip* sterile, composed of interwoven hyphae (3.5–9.5 µm wide), septate, clamped, apex subclavate to clavate, thin-walled, becoming parallel towards the apex. *Hymenophoral trama* composed of hyphae (3–7 µm wide), clamped, in subparallel to interwoven pattern. *Pileipellis* composed of densely interwoven, erect hyphae (2.5–6 µm wide), cylindrical, thin-walled, clamped, the terminal cell rounded at the apex, with pale yellow intracellular pigment. *Clamp connections* present.

Habitat—Gregarious or scattered on the ground of broad-leaved forests dominated by Ericaceae and Fagaceae.

Additional specimens examined—CHINA. Yunnan Province, Jianchuan County (剑川县), N 26°17′52″, E 99°46′9″, elev. 2954 m, 6 October 2020, on the ground of subalpine broad-leaved forests dominated by Ericaceae and Fagaceae, *Zhang Wenhao 606-1* (MHKMU WH Zhang 606-1).

Known distribution—China: Yunnan Province, elev. about 3000.

Notes—*Hydnum roseoalbum* is unique in having a whitish pileus, pale pink spines, 2–5-spored basidia, erectly arranged hyphae in the pileipellis and globose basidiospores. This taxon is closely related to *H. tomaense* R. Sugaw. & N. Endo from Japan and *H. treui* Tedersoo, Liimat. & Niskanen from Papua New Guinea [20,22]. However, *H. tomaense* has larger basidiomata (pileus is 4–10 cm in diam.), an umbilicate pileal center, 2–4-spored basidia, smaller, narrower basidiospores (7.5–8.5 × 7–8 µm, Q = 1.07–1.12), and occurs in temperate mixed forests [22]. *Hydnum treui* differs from *H. roseoalbum* in having small-sized basidiomata (pileus 3–3.5 cm wide), non-decurrent, whitish spines, 2–4-spored basidia with short sterigmata (2.5–3.5 μm), smaller basidiospores (5.5–7.0 × 5.5–7.0 µm), and a tropical distribution [20]. Furthermore, *H. roseoalbum* is similar to *H. boreorepandum* Niskanen, Liimat. & Niemelä from Finland and *H. repandum* from Sweden morphologically [20]. Nevertheless, *H. boreorepandum* has larger basidiomata (pileus up to 9 cm in diam.), whitish to creamy spines, 4–5-spored basidia, smaller basidiospores (7.0–8.5 × 6.2–7.5 μm), and an occurrence in coniferous forests [20]. *Hydnum repandum* can be distinguished by larger basidiomata (pileus up to 11 cm in diam.), creamy spines, 4-spored basidia, smaller and narrower basidiospores (7.0–8.5 × 6.2–7.5 μm), and occurs in mixed forests [20].

**Figure 9:**
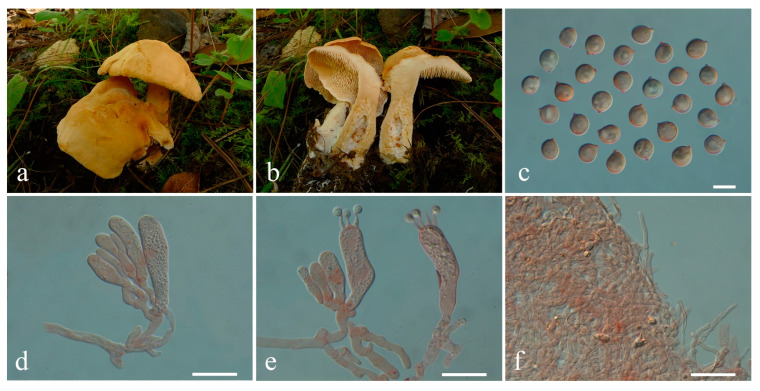
Macroscopic and microscopic features of *Hydnum roseotangerinum* from MHKMU LP Tang 3458 (Holotype). (**a**,**b**) Basidiomata; (**c**) Basidiospores; (**d**) Basidioles; (**e**) Basidia; (**f**) Pileipellis. Bars (**c**) = 10 μm; (**d**,**e**) = 20 μm; (**f**) = 50 μm.

***Hydnum roseotangerinum*** L.P. Tang & L.J. Su sp. nov. (Figure 9a–f)

Chinese name—粉橙齿菌

MycoBank—MB 855837

Etymology—Latin ‘*roseo*’ = pink, ‘*tangerinum*’ = orange, refers to the color of spines.

Diagnosis—Distinct from other species within *Hydnum* by its brownish orange pileus, pinkish orange spines, globose to subglobose, sometimes broadly ellipsoid basidiospores, and occurrence in mixed subalpine forests.

Holotype—CHINA. Yunnan Province, Yi Family Village (彝家村), N 27°23′14″, E 100°06′31″, elev. 3170 m, 26 August 2020, on the ground of subalpine mixed forests dominated by *Picea likiangensis*, *Pinus armandi*, *P. densata*, *Quercus guyavifolia*, and *Rhododendron decorum*, *Tang Liping 3458* (MHKMU LP Tang 3458). GenBank accession numbers: ITS = PQ287675, nrLSU = PQ287756, *tef1* = PQ295849.

Description—*Basidiomata* medium-sized. *Pileus* 6.5 cm in diam., plano-convex; surface dry, irregularly bumpy, non-staining, azonate, light yellow to pale brownish-orange (5B6–7), with brownish orange (5C7–8) patches; margin entire, incurved. *Context* 0.9 cm thick in pileal center, fleshy, white to pale orange (5A3–5), becoming light reddish-brown on exposure. *Stipe* 0.5 cm long, 0.1 cm in diam., central to eccentric, cylindrical, white to warm cream, solid interior. *Spines* 0.4–0.5 cm long, adnexed, non-decurrent, not flattened, with an acute (rarely subacute) apex, crowded, yellowish orange (4A3) to pinkish orange (6A3–4), gradually more yellow when drying. *Odor* and *Taste* not recorded.

*Basidiospores* [80/3/2] 8.6–9.5 (–10.0) × 7.6–9.0 µm, Q = 1.00–1.18 (–1.25), Qm = 1.08 ± 0.06, globose to subglobose, sometimes broadly ellipsoid, thin-walled, inamyloid. *Basidia* 38–58 × 8–12.5 µm, subcylindric to clavate, 2- to 4-spored, clamped; sterigmata 3.5–9 µm long. *Basidioles* numerous, subcylindrical or subclavate, smaller than basidia. *Cystidia* absent. *Spine tip* sterile, composed of interwoven hyphae (5–11.5 µm wide), septate, clamped, apex subclavate to clavate, thin-walled, becoming parallel towards the apex. *Hymenophoral trama* composed of hyphae (2–6 µm wide), clamped, in subparallel to interwoven pattern. *Pileipellis* composed of densely interwoven, subparallel to suberect hyphae (3.5–9.5 µm wide), a cylindrical, thin-walled, clamped, the terminal cell rounded at the apex, with a pale yellowish-brown intracellular pigment. *Clamp connections* present.

Habitat—Solitary or concrescent on the ground of mixed forests dominated by Ericaceae, Fagaceae, and Pinaceae.

Additional specimens examined—CHINA. Yunnan Province, Yi Family Village (彝家村), N 27°231′4″, E 100°06′31″, elev. 3170 m, 26 August 2020, on the ground of subalpine mixed forests dominated by *Picea likiangensis*, *Pinus armandi*, *P. densata*, *Quercus guyavifolia*, and *Rhododendron decorum*, *Tang Liping 3458-1* (MHKMU LP Tang 3458-1).

Known distribution—China: Yunnan Province, elev. about 3100 m.

Notes—*Hydnum roseotangerinum* is unique due to its yellow to pale brownish-orange pileus and spines, reddened or darkened context on exposure, 2–4-spored basidia, and globose to subglobose basidiospores. Morphologically, *H. roseotangerinum* is similar to *H. melitosarx* Ruots., Huhtinen, Olariaga, Niskanen, Liimat. & Ammirati, *H. ovoideisporum* Olariaga, Grebenc, Salcedo & M.P. Martín, and *H. ventricosum*. *Hydnum melitosarx* was originally described from the USA and also reported in China [20,21,26]. However, this taxon has whitish spines, three-spored basidia, and small basidiospores (7.0–8.6 × 6.4–7.8 μm) [20,26]. *Hydnum ovoideisporum* described from Spain has a pileus covered with small, erect scales, 4–5-spored basidia, and ovoid to broadly ellipsoid basidiospores (Q = 1.27–1.38) [28]. The Chinese species, *H. ventricosum*, is also closely related to *H. roseotangerinum*, but the former differs by its thin context (1–2.5 mm thick), orange-white spines, 2–4-spored basidia, and subglobose, smaller basidiospores (8.2–9.0 × 7.5–8.5 μm) [18]. Furthermore, *H. roseotangerinum* is closely related to *H. berkeleyanum* phylogenetically, but the latter is distinguished by more robust basidiomata (pileus up to 8 cm in diam.), a brown pileal center, longer spines (9 mm), and 2–4-spored basidia [24].

**Figure 10:**
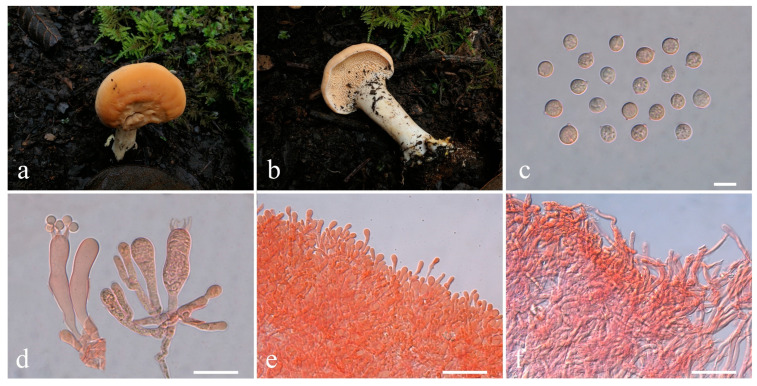
Macroscopic and microscopic features of *Hydnum sphaericum* from MHKMU X Na 149. (**a**,**b**) Basidiomata; (**c**) Basidiospores; (**d**) Basidia; (**e**) Basidioles; (**f**) Pileipellis. Bars (**c**) = 10 μm; (**d**) = 20 μm; (**e**,**f**) = 50 μm.

***Hydnum sphaericum*** T. Cao & H.S. Yuan, Stud. Mycol. 99: 100121, 2021 (Figure 10a–f)

Chinese name—圆盖齿菌

MycoBank—MB 839420

Description—Morphological characteristics are described in detail by Cao et al. [18].

Specimens examined—CHINA. Yunnan Province, Yulong County (玉龙县), N 26°41′52′′, E 100°02′09′′, elev. 3194 m, 21 August 2020, on the ground of subalpine mixed forests dominated by *Picea asperata* Mast and *Pinus densata*, *Na Xin 149* (MHKMU N Na 149).

Known distribution—China: Hunan [18], elev. unknown, and Yunnan Province, elev. above 3000 m (this study).

Notes—This species was originally described from Hunan Province, and the distribution in Yunnan Province was also confirmed in this study. So far, this species is found in central and southwestern China and grows in mixed broad-leaved and coniferous forests [18].

**Figure 11:**
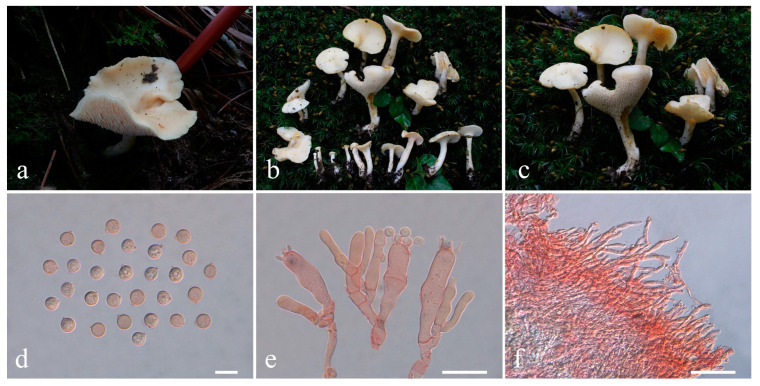
Macroscopic and microscopic features of *Hydnum tenuistipitum*. (**a**–**c**) Basidiomata, (**a**) from MHKMU LP Tang 2116, (**b**, **c**) from MHKMU HY Huang 151; (**d**) Basidiospores; (**e**) Basidia; (**f**) Pileipellis. Bars (**d**) = 10 μm; (**e**) = 20 μm; (**f**) = 50 μm.

***Hydnum tenuistipitum*** T. Cao & H.S. Yuan, Stud. Mycol. 99: 100121, 2021 (Figure 11a–f)

Chinese name—细柄齿菌

MycoBank—MB 839422

Description—Morphological characteristics are described in detail by Cao et al. [18].

Specimens examined—CHINA. Yunnan Province, Tengchong City (腾冲市), N 25°19′58″, E 98°35′30″, elev. 1710 m, 1 August 2015, *Tang Liping 2116* (MHKMU LP Tang 2116), on the ground of subtropical mixed forests dominated by *Pinus armandi*; in the same location, N 25°19′58″, E 98°35′30″, elev. 1710 m, 1 August 2015, *Zhao Jie 225* (MHKMU J Zhao 225), on the ground of subtropical mixed forests dominated by *P. armandi*; Shizong County (师宗县), N 24°38′57″, E 104°9′57″, elev. 2269 m, 23 August 2018, *Huang Hongyan 151* (MHKMU HY Huang 151), on the ground of broad-leaved forests dominated by *Quercus* spp., Ericaceae and Juglandaceae.

Known distribution—China: Hunan [18], elev. unknown, and Yunnan Province, elev. 1700–2300 m (this study).

Notes—*Hydnum tenuistipitum* was originally described from Hunan Province, and our phylogenetic analyses also confirmed its distribution in Yunnan Province. Up to now, this species has been found in mixed forests dominated by *Pinus armandi* and *Quercus* spp. in central and southwestern China [18].

**Figure 12:**
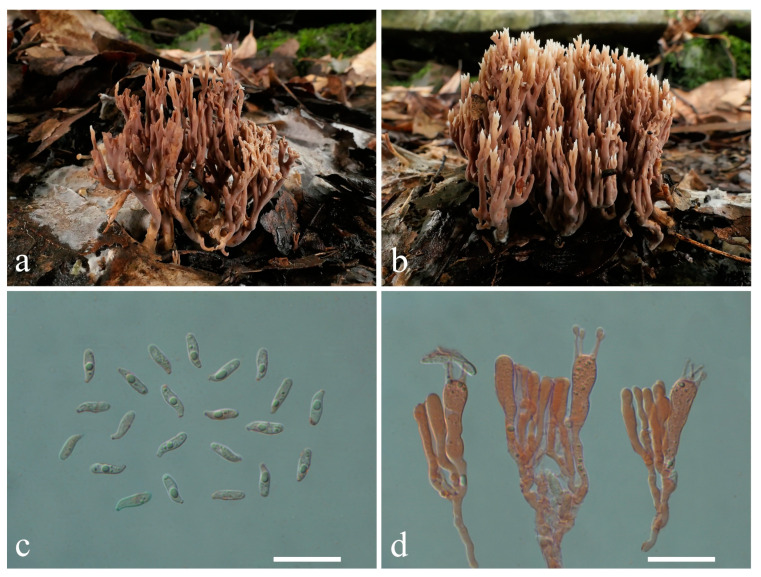
Macroscopic and microscopic features of *Lentaria bambusina* from MHKMU C Xu 071. (**a**,**b**) Basidiomata; (**c**) Basidiospores; (**d**) Basidia. Bars (**c**,**d**) = 20 μm.

***Lentaria bambusina*** P. Zhang & Zuo H. Chen, Mycol. Progr. 16(6): 606, 2017 (Figure 12a–d)

Chinese name—竹林木瑚菌 [56]

MycoBank—MB 817995

Description—*Basidiomata* cespitose or gregarious, 2.8–3.3 cm high, 3 cm wide, branched in 3–4 ranks, light brown when young, becoming brown to brownish gray when maturity, arising from a felty white mycelia layer permeating the substratum. *Stipe* solitary or conjunct, irregularly rounded, brownish gray, often covered by a felty, creamy mycelial layer, and divided into 3–4 major branches, pliable. *Major branches* erect, irregularly rounded in cross-section, concolorous to stipe, surface smooth. *Branches* erect, lax, diminishing gradually upward, elastic, surface smooth, slightly paler than major branches, light brown, subapical pale ochraceous; apices prolonged, awl-shaped, creamy. *Taste* and *odor* not recorded.

*Rhizomorph hyphae* 1.5–5 μm in diam., with common ampulliform connections swollen up to 12 μm, thin- to slightly thick-walled (wall up to 0.5 μm thick), conspicuously clamped, tightly packed. *Branch tramal hyphae* 2.5–5 μm in diam., hyaline, thin- to slightly thick-walled (wall up to 0.5 μm thick), with common ampulliform clamp connections swollen up to 7.5 μm, parallel. *Subhymenium* composed of clamped hyphae 2–4 μm in diam. *Hymenium* thickening; *Basidia* 34.5–49 × 6–8 μm, clavate, clamped, thin-walled; sterigmata 4, 4–7.5 μm long. *Cystidia* none. *Basidiospores* [40/2/1] 10.0–11.4 × 2.8–3.3 μm, Q = 3.03–3.76 (−3.93), Qm = 3.48 ± 0.25], elongated pip-shaped to cylindrical, sway-back, rounded and blunt at the apex, subacute at the base, smooth, colorless, hyaline, thin-walled, non-amyloid; hilar appendix gradual, inconspicuous.

Habitat—Cespitose or gregarious on the ground of mixed forests dominated by Fagaceae, Pinaceae, and Poaceae (Bamboo).

Specimens examined—CHINA. Anhui Province, Shitai County (石台县), Fengxingwan (凤形湾), N 30°00′20″, E 117°27′′98″, elev. 955 m, 21 July 2023, on the ground of subtropical mixed forests dominated by Fagaceae, *Xu Chang 071* (MHKMU C Xu 071).

Known distribution—China: Anhui (this study) and Hunan Province [31], elev. 600–1000 m.

Notes—*Lentaria bambusina* differs from other species due to its brown to dark brown basidiomata and elongated, pip-shaped to cylindrical basidiospores. The epithet ‘*bambusina*’ comes from its habitat under bamboo forests in the original description [31]. According to our collections, this species can also be found in mixed forests dominated by Fagaceae. This means *L. bambusina* can grow on a variety of plant substrates. At present, this taxon is found in central China (Hunan Province) and eastern China (Anhui Province). The macroscopic morphology of our collections is consistent with the original description, but the basidiospores are slightly shorter than the type (9.5–13 × 2.5–3.5 μm) [31].

**Figure 13:**
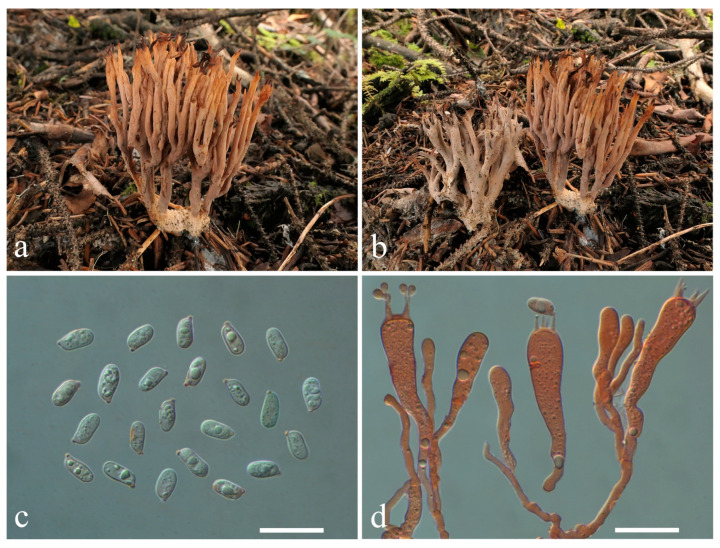
Macroscopic and microscopic features of *Lentaria subalpina* from MHKMU TJ Yu 206 (Holotype). (**a**,**b**) Basidiomata; (**c**) Basidiospores; (**d**) Basidia. Bars (**c**,**d**) = 20 μm.

***Lentaria subalpina*** L.P. Tang, L.J. Su & T.J. Yu sp. nov. (Figure 13a–d)

Chinese name—亚高山木瑚菌

MycoBank—MB 855850

Etymology—The Latin ‘*subalpina*’ refers to this species growing in subalpine forests.

Diagnosis—Distinct from other *Lentaria* species mainly due to the grayish orange to brownish orange basidiomata, sometimes carbonized, black branch apices, elongated ellipsoid to cylindrical basidiospores (9.0–11.0 × 4.8–5.7 μm), and growing in dead branches of *Abies* or *Picea* in subalpine forests.

Holotype—CHINA. Yunnan Province, Ninglang County (宁蒗县), Gewa Village (格瓦村), N 27°43′21″, E 100°31′′54″, elev. 3468 m, 2 October 2023, growing in dead branches of *Abies fabri* (Mast.) Craib or *Picea asperata* in subalpine mixed forests, *Yu Taijie 206* (MHKMU TJ Yu 206). GenBank accession numbers: ITS = PQ287677, nrLSU = PQ287758.

Description—*Basidiomata* cespitose or gregarious, 3.5–5 cm high, 2 cm wide, branched in 3–4 ranks, pale orange (6B3–5) when young, becoming grayish orange (5B3), brownish orange (6C3–4) to brownish gray (6C2) when mature, arising from a felty white mycelia layer permeating substratum. *Stipe* solitary or conjunct, up to 2.0 × 0.5 cm, irregularly rounded, brownish orange to brownish gray, often covered by a felty, creamy mycelial layer, and divided into 2–3 major branches, pliable. *Major branches* erect, irregularly rounded in cross-section, somewhat darker than stipe, brownish orange to brownish gray, surface smooth. *Branches* erect, lax, diminishing gradually upward, elastic, surface smooth, concolorous to major branches, subapical pale orange; apices prolonged, awl-shaped, carbonized due to dryness. *Taste* and *odor* not recorded.

*Rhizomorph hyphae* 2.4–4 μm in diam, thin- to slightly thick-walled (wall up to 0.5 μm thick), conspicuously clamped, tightly packed. *Branch tramal hyphae* 2.5–5 μm in diam., hyaline, thin- to slightly thick-walled (wall up to 0.5 μm thick), with common ampulliform clamp connections swollen up to 9.5 μm, parallel. *Subhymenium* composed of clamped hyphae 2.5–3.6 μm in diam. *Hymenium* thickening; *Basidia* 40–74 × 8–10.5 μm, clavate, clamped, thin-walled; sterigmata 4, 4–8 μm long. *Cystidia* none. *Basidiospores* [80/2/2] 9.0–11.0 (−11.4) × (4.3–) 4.8–5.7 (−6.7) μm, Q = (1.53–) 1.64–2.29 (−2.44), Qm = 1.96 ± 0.19, elongated ellipsoid to broadly cylindrical, rounded and blunt at the apex, subacute at the base, smooth, colorless, hyaline, thin-walled, non-amyloid; hilar appendix gradual, inconspicuous.

Habitat—Cespitose or gregarious, growing in dead branches of *Abies* or *Picea* in subalpine mixed forests.

Additional specimens examined—Paratype: CHINA. Yunnan Province, Ninglang County (宁蒗县), Gewa Village (格瓦村), N 27°43′21″, E 100°31′54″, elev. 3468 m, 2 October 2023, growing in dead branches of *Abies fabri* or *Picea asperata*, *Xia Xing 129* (MHKMU X Xia 129).

Known distribution—China: Yunnan Province, elev. about 3400 m.

Notes—*Lentaria subalpina* has orange to brownish basidiomata, large basidia, broadly cylindrical basidiospores, grows in dead wood of gymnospermous plants, such as *Abies* or *Picea* in subalpine forests, and is only found in subalpine regions of Yunnan Province, China. This species is characterized by its orange-brown basidiomata, robust branches, larger basidia, and elongated ellipsoid to broadly cylindrical basidiospores. *Lentaria* s.l. species always have high length/width ratios of basidiospores, and species of this genera vary greatly in size [30,31]. In morphology, *L. subalpina* is similar to *L. bambusina*, but the latter differs in having darker basidiomata, smaller basidia, longer basidiospores, and association with angiospermous plants, such as bamboo or broad-leaved trees [31]. Furthermore, *L. subalpina* is closely related to *L. byssiseda* and *L. micheneri* phylogenetically. However, *L. byssiseda* is distinguished by having white, *Ramaria*-like basidiomata, larger basidiospores (12–15.5 × 3.5–4.5 μm), and a distribution in north temperate regions [31,34]; *L. micheneri* has dark basidiomata (brown to chocolate brown), small, short-cylindrical basidiospores (6.5–7.6 × 4.0–4.3 μm), and occurs in the USA [30,32]. Another species, *L. uncispora* P. Zhang & Zuo H. Chen, also occurs on rotten stumps of fir covered by moss in the high-altitude zone of Sichuan, China. But it has fleshy off-white to fleshy pink basidiomata, looking like *Ramaria*, and has longer basidiospores (24–27 × 3.5–4 μm) [31].

**Figure 14:**
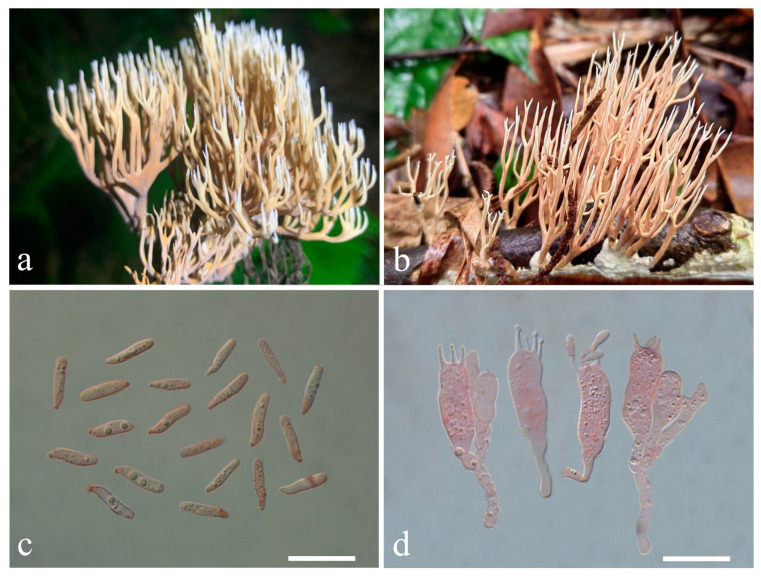
Macroscopic and microscopic features of *Lentaria surculus* from MHKMU LJ Su 430. (**a**,**b**) Basidiomata, (**a**) photographed by Haipu Fu, (**b**) photographed by Weiliang Wang; (**c**) Basidiospores; (**d**) Basidia. Bars (**c**,**d**) = 20 μm.

***Lentaria surculus*** (Berk.) Corner, Ann. Bot. Mem. 1: 444. 1950. (Figure 14a–d)

Chinese name—枝木瑚菌 [57]

Description—*Basidiomata* cespitose or gregarious, 3–6 cm high, 3–5 cm wide, branched in 3–5 ranks, light yellow, yellowish brown to brown, arising from a felty white mycelia layer permeating substratum. *Stipe* solitary or conjunct, up to 1.5–2 × 0.5–0.7 cm, irregularly rounded, light brown to brown, color becomes darker after injury, often covered by a felty, creamy mycelial layer and divided into 3–5 major branches, pliable. *Major branches* erect, irregularly rounded in cross-section, somewhat darker than stipe, yellowish brown to brown, surface smooth. *Branches* erect, lax, diminishing gradually upward, elastic, surface smooth, slightly paler than major branches, light yellow to light brown; apices prolonged, awl-shaped, white. *Taste* and *odor* not recorded.

*Rhizomorph hyphae* 2–4.5 μm in diam, with common ampulliform connections swollen up to 6.5–15 μm, thin- to slightly thick-walled (wall up to 0.5 μm thick), conspicuously clamped, tightly packed. *Branch tramal hyphae* 3–7.5 μm in diam., hyaline, thin- to slightly thick-walled (wall up to 0.5 μm thick), with common ampulliform clamp connections swollen up to 7.5–12 μm, parallel. *Subhymenium* composed of clamped hyphae 4.5–7.5 μm in diam. *Hymenium* thickening; *Basidia* 37–50 × 7.8–10.5 μm, clavate, clamped, thin-walled; sterigmata 3.5–9 μm long. *Cystidia* none. *Basidiospores* [80/2/2] (12.2–) 13.5–17.8 (−19.6) × 3.0–3.9 (−4.3) μm, Q = (3.56–) 3.74–5.23 (−6.53), Qm = 4.45 ± 0.55, cylindrical to elongated cylindrical, rounded and blunt at the apex, subacute at the base, smooth, colorless, hyaline, thin-walled, non-amyloid; hilar appendix gradual, inconspicuous.

Habitat—Cespitose or gregarious, growing in dead branches in tropical forests.

Specimens examined—CHINA. Hainan Province, Lanyang Town (兰洋镇), Fanjia Village (番加村), N 19°17′52″, E 109°41′′20″, elev. 245 m, 15 October 2024, growing in dead branches of *Hevea brasiliensis* (Willd. ex A. Juss.) Müll. Arg., *Su Linjie* 430 (MHKMU LJ Su 430), collected by Haipu Fu; in the same location, N 19°17′52″, E 109°41′′20″, elev. 245 m, 15 October 2024, *Su Linjie* 431 (MHKMU LJ Su 431), collected by Haipu Fu.

Known distribution—China: Guangdong [31], elev. unknown, Hainan (this study), elev. about 250 m, and Yunnan Province [31], elev. unknown; Argentina, Bhutan, Bolivia, Brazil, Jamaica, Malaysia, Philippines (type location), Uganda, and United States [34], elev. unknown.

Notes—*Lentaria surculus* is characterized by its paler-colored, slender, and pliable basidiomata and a high spore length/width ratio. It was originally described from the Philippines and subsequently reported from Africa, America, and tropical Asia [34,58]. This species is a typical tropical species with a wide distribution around the world. In China, this species is distributed in Guangdong, Hainan, and tropical regions of Yunnan Province [31,58].

## 4. Discussion

In this study, five new species, viz. *H. cremeum*, *H. flavosquamosum*, *H. roseoalbum*, *H. roseotangerinum*, and *L. subalpina*, were proposed based on morphological and molecular analyses. Additionally, our results added new information on three known species, *H. berkeleyanum*, *L. bambusina*, and *L. surculus*, and *H. flabellatum* was proposed as a synonym of *H. pallidomarginatum*.

In the genus *Hydnum*, there remain several unidentified taxa in China. For example, *H. vesterholtii* Olariaga, Grebenc, Salcedo & M.P. Martín originated from France and is also reported in China [19,26]. However, our phylogenetic analysis revealed that Chinese specimens labeled ‘*H. vesterholtii*’ were divided into several different lineages, such as MHKMU LP Tang 2886 (*Hydnum* sp. 3) and HKAS92343. However, none of them were grouped with the type specimen of *H. vesterholtii*. Therefore, we infer that *H. vesterholtii* may not be present in China, and Chinese specimens likely represent at least three under-descried taxa. To date, there are 24 species within the genus *Hydnum* distributed in China, and 13 of them are found in Yunnan Province [18,21,26]. *Hydnum minum* Yanaga & N. Maek. was described from Japan by Yanaga et al. [25]; then, it was assigned to the subgen. *Alba* s. l. by Niskanen et al. [20]. However, Qin et al. transferred it to the subgen. *Brevispina*, and they removed *H. tenuistipitum* and *H. microcarpum* Ming Zhang from the subgen. *Brevispina* [21]. Nevertheless, our result was consistent with Niskanen et al. [20] and Zhang et al. [26], indicating that *H. minum* emerged into the subgen. *Alba* s.l. rather than the subgen. *Brevispina*, with *H. tenuistipitum* and *H. microcarpum* still located in the subgen. *Brevispina*. Qin et al. used only two loci, ITS and nrLSU, to conduct their phylogenetic analysis [21]. However, the nrLSU was poor at discriminating related species of this genus, while the *tef1* locus is suitable for related species delimitation [18,19,22,48]. Therefore, the addition of *tef1* sequences made the results more accurate. In addition, it is necessary to tightly integrate molecular data, morphological characteristics, distribution areas, habitats, etc. in analyzing.

A new taxon from the genus *Lentaria* s.l. was described, and it is the first species originating from southwestern China. To date, six species within the genus *Lentaria* s.l. are reported in China, and two of them are found in Yunnan [31]. *Lentaria* s.l. species tend to grow on specific substrates. Typically, temperate and subalpine species grew on gymnosperm rotting wood, and subtropical and tropical species grew on angiosperm rotting wood [31,33]. As mentioned by Liu et al. [31] and in our results, the genus *Lentaria* s.l. consists of two main clades, and this difference is consistent with variations in distribution and substrate preference. Nevertheless, there are almost exclusive ITS sequences in the database and just a few of them are useful, which results in the phylogenetic relationships of *Lentaria* s.l. being difficult to be resolved in-depth. To further reveal the interspecific relationships of *Lentaria* s.l. species with related fungal taxa, more materials and suitable loci should be incorporated in the molecular phylogenetic analysis.

**Key to species of *Lentaria* s.l. in China**

1. Average length of basidiospores more than 18 μm.............................................................*Lentaria uncispora*
1. Average length of basidiospores less than 18 μm...........................................................................................2
2. Temperate or subtropical–subalpine distribution, growing on rotten branches of coniferous trees........3
2. Tropical or subtropical distribution, growing on rotten branches of broad-leaved trees..........................5
3. Basidiomata grayish- to brownish-orange.....................................................................................*L. subaplina*
3. Basidiomata off-white, buff to pale tan............................................................................................................4
4. Average length of basidiospores more than 9 μm.........................................................................*L. byssiseda*
4. Average length of basidiospores less than 9 μm.......................................................................*L. patouillardii*
5. The basidiomata robust, basidiospores smaller (10.0–11.4 × 2.8–3.3 μm).................................*L. bambusina*
5. The basidiomata slender, basidiospores larger (13.5–17.8 × 3.0–3.9 μm).....................................*L. surculus*

## Figures and Tables

**Table 1:** Information of specimens examined in this study.

No.	Specimen Voucher	Locality	Habitat	Altitude	Longitude and Latitude	Date
1	MHKMU C Xu 071	China: Anhui	mixed forests	955 m	N 30°00′20″, E 117°27′98″	21 July 2023
2	MHKMU HY Huang 151	China: Yunnan	broad-leaved forests	2269 m	N 24°38′57″, E 104°09′57″	23 August 2018
3	MHKMU HY Huang 873	China: Yunnan	mixed forests	2731 m	N 27°21′16″, E 100°09′37″	24 August 2020
4	MHKMU J Zhao 225	China: Yunnan	mixed forests	1710 m	N 25°19′58″, E 98°35′30″	1 August 2015
5	MHKMU LJ Su 430	China: Hainan	broad-leaved forests	245 m	N 19°17′52″, E 109°41′20″	15 August 2024
6	MHKMU LJ Su 431	China: Hainan	broad-leaved forests	245 m	N 19°17′52″, E 109°41′20″	15 August 2024
7	MHKMU LP Tang 2116	China: Yunnan	mixed forests	1710 m	N 25°19′58″, E 98°35′30″	1 August 2015
8	MHKMU LP Tang 2886	China: Jilin	mixed forests	820 m	N 42°22′19″, E 128°00′15″	20 August 2019
9	MHKMU LP Tang 3319	China: Yunnan	mixed forests	2890 m	N 26°46′11″, E 100°02′51″	21 August 2020
10	MHKMU LP Tang 3453	China: Yunnan	mixed forests	3170 m	N 27°23′14″, E 100°06′31″	26 August 2020
11	MHKMU LP Tang 3454	China: Yunnan	mixed forests	3170 m	N 27°23′14″, E 100°06′31″	26 August 2020
12	MHKMU LP Tang 3458	China: Yunnan	mixed forests	3170 m	N 27°23′14″, E 100°06′31″	26 August 2020
13	MHKMU LP Tang 3458-1	China: Yunnan	mixed forests	3170 m	N 27°23′14″, E 100°06′31″	26 August 2020
14	MHKMU M Mu 740	China: Yunnan	mixed forests	3180 m	N 26°58′26″, E 100°10′47″	22 August 2020
15	MHKMU M Mu 791	China: Yunnan	mixed forests	3240 m	N 27°23′14″, E 100°06′19″	26 August 2020
16	MHKMU SD Yang 378	China: Yunnan	mixed forests	2130 m	N 25°36′51″, E 102°09′23″	20 August 2016
17	MHKMU SD Yang 557	China: Yunnan	broad-leaved forests	2205 m	N 24°39′27″, E 104°10′17″	24 August 2018
18	MHKMU TJ Yu 197	China: Yunnan	mixed forests	3116 m	N 27°42′34″, E 100°31′30″	2 October 2023
19	MHKMU TJ Yu 206	China: Yunnan	mixed forests	3468 m	N 27°43′21″, E 100°31′54″	2 October 2023
20	MHKMU WH Zhang 599	China: Yunnan	broad-leaved forests	2500 m	N 26°23′41″, E 99°50′10″	5 October 2020
21	MHKMU WH Zhang 606	China: Yunnan	broad-leaved forests	2954 m	N 26°17′52″, E 99°46′09″	6 October 2020
22	MHKMU WH Zhang 606-1	China: Yunnan	broad-leaved forests	2954 m	N 26°17′52″, E 99°46′09″	6 October 2020
23	MHKMU X Na 149	China: Yunnan	mixed forests	3194 m	N 26°41′52″, E 100°02′09″	21 August 2020
24	MHKMU X Xia 129	China: Yunnan	mixed forests	3468 m	N 27°43′21″, E 100°31′54″	2 October 2023
25	MHKMU YJ Pu 361	China: Yunnan	mixed forests	3180 m	N 26°58′26″, E 100°10′47″	23 August 2020
26	MHKMU YR Li 004	China: Yunnan	mixed forests	2090 m	N 25°36′51″, E 102°09′23″	20 August 2016

## Data Availability

The datasets presented in this study can be found in online repositories. The accession number(s) can be found in GenBank (https://www.ncbi.nlm.nih.gov/genbank/, accessed on 7 September 2024), MycoBank (https://www.mycobank.org/, accessed on 21 September 2024), and Treebase (https://treebase.org/, accessed on 15 October 2024).

## Supplementary Materials

The following supporting information can be downloaded at https://www.mdpi.com/article/10.3390/jof10120824/s1. Figure S1. Phylogenetic tree of genus *Hydnum* based on ITS dataset; Figure S2. Phylogenetic tree of genus *Hydnum* based on nrLSU dataset; Figure S3. Phylogenetic tree of genus *Hydnum* based on *tef1* dataset; Figure S4. Phylogenetic tree of genus *Lentaria* based on ITS dataset.
