# Supplementary material for: New Contributions on Species Diversity of Genus Hydnum and Lentaria s.l. in China
Source: J Fungi (Basel). 2024 Nov 27;10(12):824. doi: 10.3390/jof10120824 (PMC11678670; doi:10.3390/jof10120824)
Supplement: Supplementary file 1 [file jof-10-00824-s001.zip › jof-3302173-supplementary.pdf]

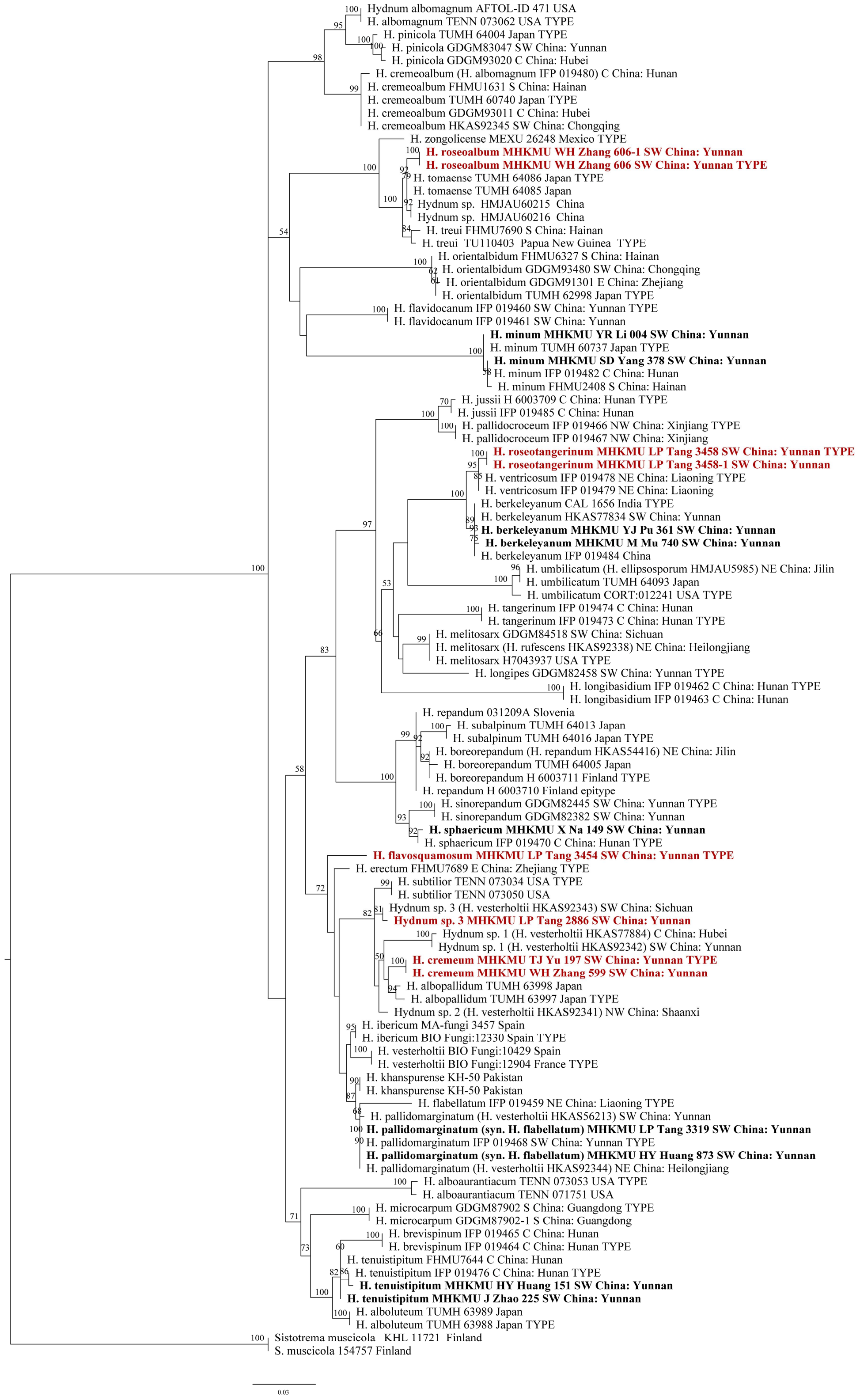

Figure S1. Phylogenetic tree of genus *Hydnum* based on ITS dataset.

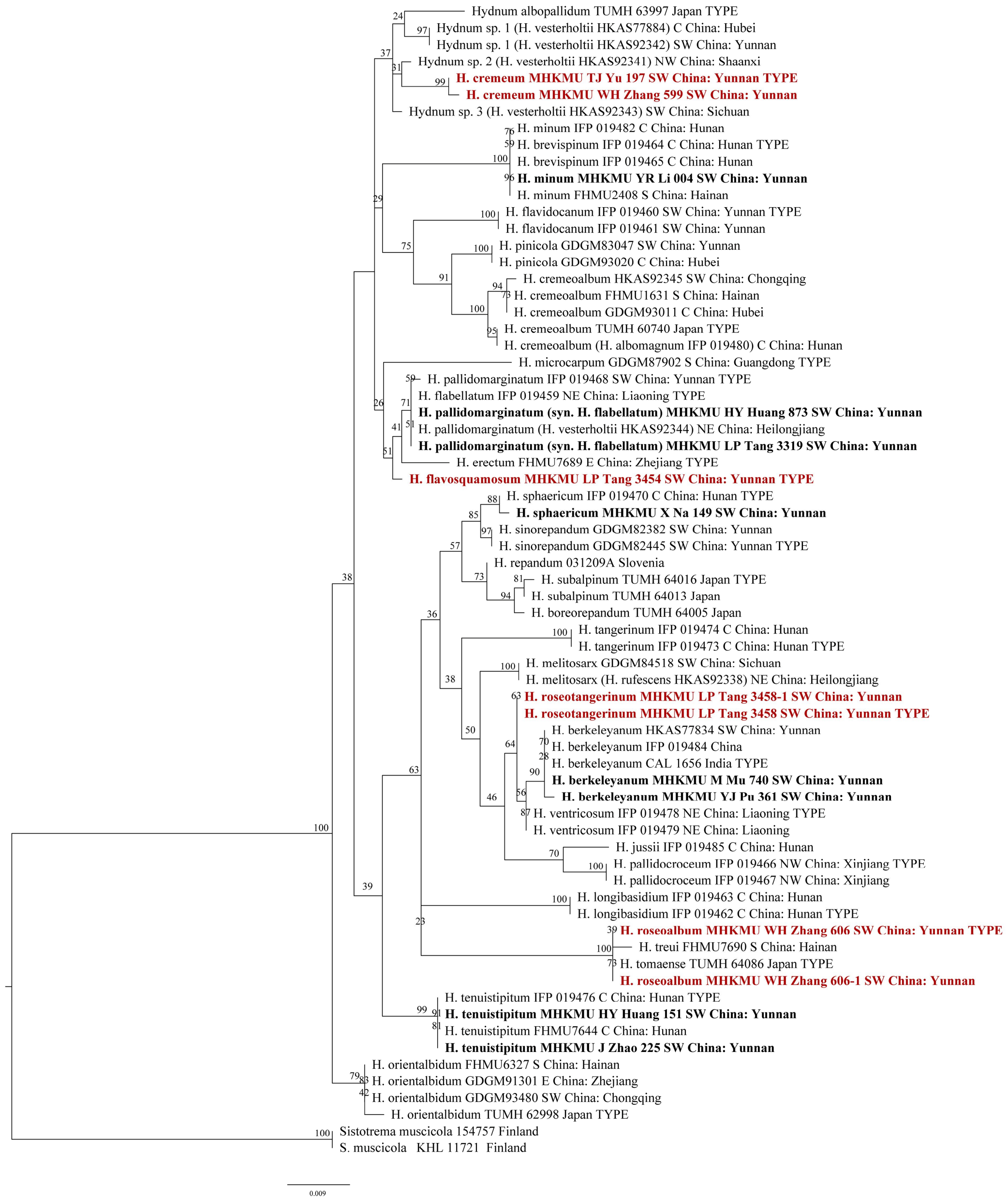

Figure S2. Phylogenetic tree of genus *Hydnum* based on nrLSU dataset.

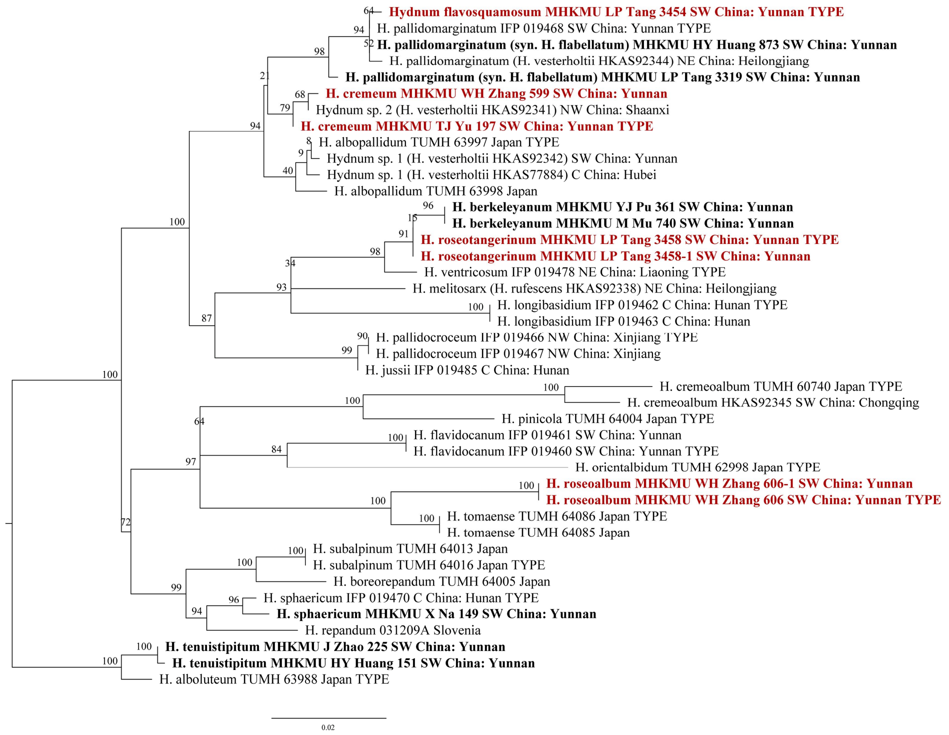

Figure S3. Phylogenetic tree of genus *Hydnum* based on *tef1* dataset.

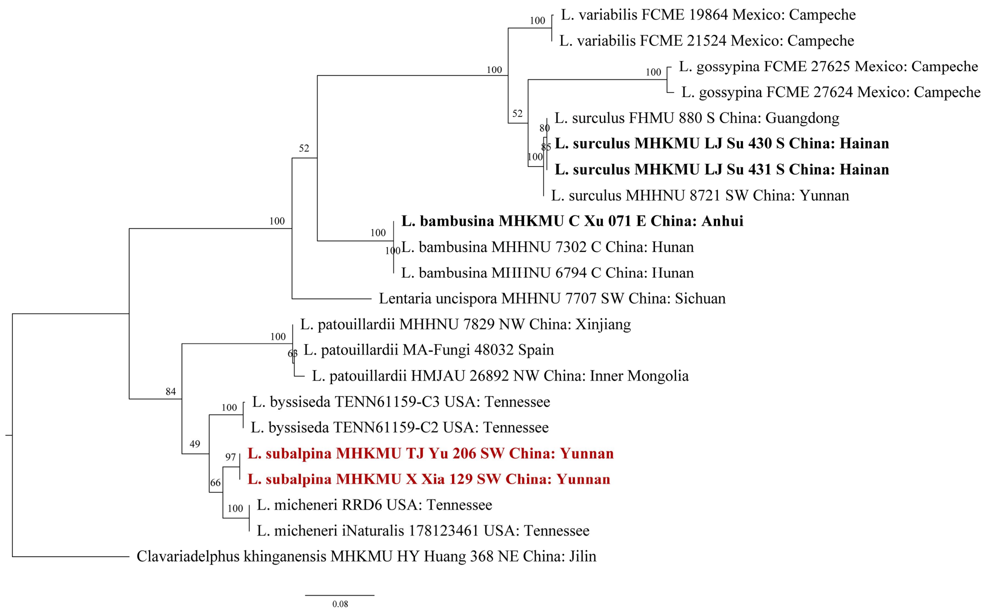

Figure S4. Phylogenetic tree of genus *Lentaria* based on ITS dataset.
